# Iterative Variable Gene Discovery from Whole Genome Sequencing with a Bootstrapped Multiresolution Algorithm

**DOI:** 10.1155/2019/3780245

**Published:** 2019-02-11

**Authors:** David N. Olivieri, Francisco Gambón-Deza

**Affiliations:** ^1^Department of Computer Science, University of Vigo, Ourense 32004, Spain; ^2^Department of Immunology, Hospital of Meixoeiro, Vigo, Spain

## Abstract

In jawed vertebrates, variable (V) genes code for antigen-binding regions of B and T lymphocyte receptors, which generate a specific response to foreign pathogens. Obtaining the detailed repertoire of these genes across the jawed vertebrate kingdom would help to understand their evolution and function. However, annotations of V-genes are known for only a few model species since their extraction is not amenable to standard gene finding algorithms. Also, the more distant evolution of a taxon is from such model species, and there is less homology between their V-gene sequences. Here, we present an iterative supervised machine learning algorithm that begins by training a small set of known and verified V-gene sequences. The algorithm successively discovers homologous unaligned V-exons from a larger set of whole genome shotgun (WGS) datasets from many taxa. Upon each iteration, newly uncovered V-genes are added to the training set for the next predictions. This iterative learning/discovery process terminates when the number of new sequences discovered is negligible. This process is akin to “online” or reinforcement learning and is proven to be useful for discovering homologous V-genes from successively more distant taxa from the original set. Results are demonstrated for 14 primate WGS datasets and validated against Ensembl annotations. This algorithm is implemented in the Python programming language and is freely available at http://vgenerepertoire.org.

## 1. Introduction

A hallmark of an adaptive immune system (AIS) is its ability to generate a large and specific response to foreign pathogens. This is accomplished through using a recognition machinery of two molecular structures, immunoglobulins (IGs) and T-cell (lymphocyte) receptors (TCRs). IGs and TCRs recognize an antigen (Ag) through different mechanisms. IG binds to an antigen in soluble form, while TCR binds to an antigen with the major histocompatibility complex (MHC) molecule [1, 2]. Antigen-binding sites in both the IG and TCR molecules possess similar recognition domains, called variable (V) domains. These domains are coded by V-genes.

Jawed vertebrate species contain multiple V-genes located within seven genomic loci. V-genes share a common sequence homology (either orthologous across species or paralogous due to gene duplication). Most jawed vertebrates have three loci for genes that encode the IG chains (IGH for heavy (H) chains and IGK and IGL for *κ* and *λ* chains, respectively) and four loci for genes that encode the TCR chains (TRA, TRB, TRG, and TRD coding for the TCR *α*-, *β*-, *γ*-, and *δ*-chains, respectively). In each locus, there is a variable number of each of these V-genes. To generate the immunoglobulin or TCR chains, one of these genes is brought to the proximity of the exons that encode the constant regions through a recombination process. This process is complex (since additional D and J gene sequences are involved) and is the basis for the wide diversity of these molecules, required for adaptive immunity. More details of the structure and function of these molecules are described elsewhere [2–4].

*Motivation for V-gene finder algorithm*: Knowing the detailed structure of these genes and the molecules they encode, as well as the entire repertoire that each species possess, would help to understand the evolution of the adaptive immune system. Nonetheless, these variable (V) gene repertoires have only been annotated for a few model taxa. The maturity and breadth of genome sequencing projects of >150 jawed vertebrate species provide an exciting opportunity to identify the full set of V-gene repertoires (i.e., the set of V-genes possessed by each species) across the entire jawed vertebrate kingdom.

*Context for questions in immunology*: In brief, there are several fundamental questions that a full understanding of the V-gene Ig/TCR germline repertoire would provide. First, it is not known why the number of V-genes vary so differently between species (for example, some species belonging to the Chiroptera family have >300 V-genes, while others, such as Cetacea, possess very few such genes, <30). Clearly, evolutionary mechanisms shaped these repertoires through complex relationships between the other molecules and the pathogenic environment of each species. By studying molecular phylogenetics of these genes for many mammals and reptiles, we have discovered that IGV has many short branches indicating rapid birth/death processes with a high turnover. In the TRV, branches are long and slowly proliferating, suggesting conservative and coevolutionary mechanisms. Other relations are also inferred, including correlations between distant loci and preferential bias of certain Vs, suggestive of redundancy and/or unknown function. For example, some new studies have provided evidence of preferentially biased V-gene repertoires in certain human diseases. Thus, more work is needed to understand the yet unknown mechanisms of V-genes. Having a complete picture of the V-gene repertoires across all vertebrates is the first step.

*Reason and brief description of new algorithm software*: While presenting bioinformatic annotation software is effective for identifying genes from their constituent exons, they are not reliable for detecting V-gene exons. This is because the exon boundaries of V-genes are markedly different (Section 2). To address this problem, we had developed a heuristic bioinformatics algorithm, called Vgenextractor, that uses highly conserved amino acid motif sequences to identify V-genes in Whole Genome Shotgun (WGS) sequences [5]. The software was able to identify more than 90% of V-genes in species whose chromosomes have been fully constructed and annotated. However, this algorithm had its limitations since it was ostensibly a filter for specific conserved motif sequences that are not completely universal.

Therefore, a probabilistic method based on machine learning was needed to identify V-gene sequences that lack these motifs and are more distant in homology from those known in model species. Here, we describe such an algorithm. It greatly extends our previous software in that it can iteratively *learn* to identify valid V-genes that do not possess canonical motifs and are structurally distant from those documented in the IMGT [3, 4]. In particular, the algorithm uses an iterative supervised machine learning process that starts with a small set of known and verified V-gene sequences and then successively discovers homologous *unaligned* sequences from the WGS sequencing datasets from many taxa. Upon each iteration, newly discovered V-genes are added to the training set for the next iteration. This iterative learning/discovery process terminates when the number of new sequences discovered is negligible. This process is akin to “online” or reinforcement learning and is particularly useful for discovering homologous V-genes from successively more distant taxa from the original set, as shown in Results.

### 1.1. Brief Background to Identify V-Genes in Genome Sequences

*About Genome Datasets and IG/TCR Loci*: Advances in next generation sequencing (NGS) technologies [6] have stimulated genome assembly projects for thousands of taxa across the entire tree of life [7]. Many WGS assembly datasets are available from the NCBI in the form of Fasta files consisting of assembled contigs, or for more mature projects, scaffolds, and/or fully constructed chromosomes. From a bioinformatics perspective, these Fasta files are large text files, approximately 3.1 GB (uncompressed), and can have 10k–3M independent contigs (more mature projects have less but larger contigs, or scaffolds, which are constructed by stitching together a large number of contigs (eventually forming the basis of an entire chromosome construction)). Presently, these datasets exist for >150 mammal species, 20 reptiles, >100 fish with an average genome coverage >15–20× (depending upon the sequencing technology) and N50 (>20 kbp) which is sufficient for uncovering approximately >90% of the V-gene repertoire of a species [5]. The patches of the genome assemblies that are still incomplete represent the only limiting factor for uncovering the full V-exon repertoires. With maturity of these projects, however, it can be expected that the full gene repertoires can be annotated.

*Structure of germline IG/TCR loci*: In jawed vertebrates (i.e., mammals, reptiles, fish, and birds), functional V-gene isotypes, corresponding to either Ig or TCR receptor molecules, are found in seven separate genomic loci. For immunoglobulin chains, there are three V-gene loci: one heavy chain (IGHV), and two light chains, referred to as *κ* (IGKV) and *λ* (IGLV). For the TCR chains, there are two types: *α*/*β* and *γ*/*δ*. The TCR *α*/*β* is composed of two chains (*α* and *β*), whose variable regions are coded in two loci, TRAV and TRBV, respectively. In a similar way, the variable regions of TCR *γ*/*δ* also are encoded by the loci TRGV and TRDV (the locus TRDV is found in the same chromosomal location as TRAV). The number of V-genes in each locus varies considerably between different chains and across different species. Additionally, varying numbers of pseudogenes—sequences that either contain stop codons or have alterations in their reading frame and are not functionally expressed V-genes—exist throughout these loci [8–10].

At present, the vast majority of genome sequencing projects exists either as WGS contigs or scaffolds (i.e., segments of the DNA, which have not been assembled nor associated at the chromosome level). Thus, the loci of IG and TCR of each individual V-gene must be inferred from sequence homology. From a molecular phylogenetic tree analysis, the V-genes from the same loci would belong to the same clade. This same classification could be automated with statistical machine learning, as will be shown.

*Other gene finding software for V-genes*: There are several bioinformatic software packages for automatically identifying genes [11] (for example, geneid [12]). However, these general algorithms, which are effective for identifying most genes, are not valid for discovering V-exons. The reason is that these algorithms use a general rule for the start/stop of exons with an AG/GT signals, whereas the exon boundaries of the V-exons are more complex and variable due to the need of the VDJ recombination mechanism. In the case of the V-exon, the GT motif does not mark the exon termination boundary, rather there is a CACAGTG motif, that is only partially conserved.

*Our previous algorithm: Vgenextractor*: Our previous algorithm assumed that V sequences must contain conserved sequence motifs near specific positions, i.e., when the amino acid length is >80, there exists a cysteine C between positions 15 and 28, a tryptophan (W) between positions 25 and 40, and the YYC motif (Y^*∗*^), or variants are found in the last 15 amino acids. The algorithm also takes advantage of the highly conserved canonical *recombination signal sequence* (RSS) motif. Knowing, to a very high degree, the exon structure obviates the need for applying a general (and genome wide) gene finding algorithm (e.g., mgene, Augustus, Craig, fgenesh, and geneid, others) that attempt to discover all protein coding genes, given wide variations of genomic segment types (i.e., intergenic, 5′ untranslated region (UTR) and coding exon, intron, or 3′ UTR). Instead, in-frame exons are identified between a nearly universal -AG- start motif and the RSS canonical -CAC- motif (i.e., a shortened version of the generally conserved motif).

*Results from our previous algorithm: Vgenextractor*: Predictions of our previous algorithm, VgenExtractor [5], described briefly above, provided a minimum confidence region for discovering V-genes in other species whose genomes are only partially annotated (more than 150 mammals and 12 reptiles species; public repository http://vgenerepertoire.org). While still representing incomplete immune repertoires, this large set of V-gene sequences has yielded heretofore unavailable information about the evolutionary origins of these IG and TCR repertoires. One example is the identification of ancestral clades found both in reptiles [13] and mammals, suggesting that V-genes in extant taxa are descendants of an ancestral immunoglobulin (Ig) recognition progenitor gene [14] that coincided with the rise of jawed vertebrates and has been maintained since then throughout their evolution [15]. These gene sequences also provide detailed clues of repertoire adaptation and divergence amongst orders. In primates, evolutionary conserved TCR clades were identified [16] that were later seen to exist throughout all present-day mammals [17].

Despite the success of the VgenExtractor pipeline, the method has several drawbacks. First, a class of sequences can be overlooked since it is probable that some V-gene sequences may not obey canonical amino acid (AA) motif conservation rules; Iguanidae, for example, possess V-genes that lack the canonical tryptophan at position 41 (tryp-41) [18]. The VgenExtractor algorithm produces a set of false positives, requiring a Blastp pipeline step to remove nonhomologous sequences and to classify V-gene sequences into their respective loci (i.e., IGHV, IGLV, IGKV, TRA/DV, TRBV, and TRGV). The Blastp step requires sequence alignment and depends on the completeness of the Nonreduntant (nr) protein database. These deficiencies may account for only 10% error in mammalian taxa, but for more distant orders (e.g., reptiles, birds, and bony fish), V-gene sequences may deviate substantially from their conserved brethren and not have sufficient representation in the blast NP database for ortholog determination.

**Figure 1:**
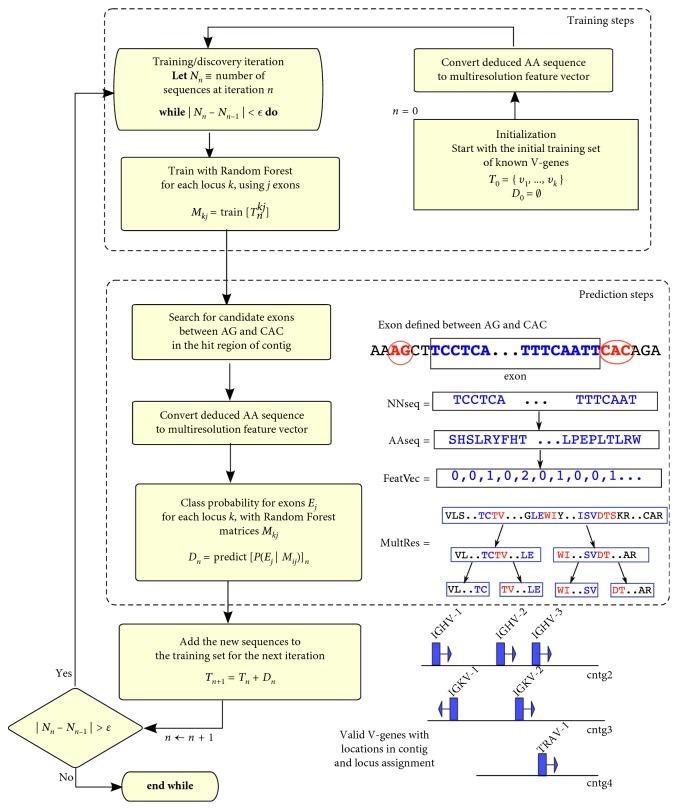
Iterative workflow for predicting V-gene repertoire from WGS datasets. The algorithm bootstraps from a small set of initial V-gene sequences (step 1); these sequences are converted from nucleotide to amino acid sequences so that a multiresolution (MR) feature vector is constructed. Random Forests are trained for each MR levels; and the training matrices are saved for each MR level. In the prediction phase, the collection of exons, obtained from different unconnected contigs the WGS files, is processed with Random Forests (for each multiresolution level) to determine those that have sufficient probability (homologous to the training sets) for being V-genes. The results are a set of V-exons classified into their respective locus.

*Difference between Vgenextractor and new ML approach*: No a priori assumptions are made about V-gene sequences, and a supervised learning algorithm was developed that starts with a known annotated set of V-genes (from humans) and iteratively discovers new sequences, gradually incorporating newly learned sequences into the next learning iteration. Such iterative algorithms are commonly applied in other machine learning tasks such as face detection, voice recognition, and natural text processing. This iterative learning methodology is termed *online* because it continually learns new information (here sequences) and thus adaptively learns in more distant situations (in this case, more V-gene sequences from more distant taxa).  Figure 1 illustrates the general iterative steps of the VgeneFinder workflow.

## 2. Methods

Here, details of the iterative algorithm are described. In particular, this includes the entire pipeline for extracting candidate exons from the WGS files, forming multiresolution feature vectors, training Random Forest classifiers, and the iterative training/prediction process. First, the genome sets used for the study are described. Next, the details of the algorithm are provided.

### 2.1. Genome Datasets

To demonstrate the iterative bootstrap learning method and validate the VgeneFinder software, 16 primates (including human) WGS datasets obtained from the NCBI were used (*M. Mulatta* was left out for validation comparisons). A detailed summary of the accession numbers and relevant assembly parameters can be found in Table 1. All WGS datasets had coverage >15× and N50 values >20k, representing an adequate threshold for identifying V-genes [13].

A listing of the primate species used (with WGS abbreviation and N50 value) are Lemuriformes: *D. madagascariensis* (AGTM01, 3.6 kbp), *O. garnettii* (AQR03, 27.1 kbp), and *M. murinus* (AAHY01, 21.7 kbp); Tarsiiformes: *T. syrichta* (ABRT01, 38.17 kbp); New World monkeys: *C. jacchus* (ACFV01, 29.3 kbp) and *S. boliviensis* (AGCE01, 38,823 kbp); Old World monkeys: *M. mulatta* (AANU01, 25.7 kbp), *M. fascicularis* (CAEC01, 8.9 kbp), *C. sabaeus* (AQIB01, 90.5 kbp), *P. anubis* (AHZZ01, 40.3 kbp); and hominids: *N. leucogenys* (ADFV01, 35.2 kbp), *P. abelii* (ABGA01, 15.6 kbp), *G. gorilla* (CABD02), *P. paniscus* (AJFE01,66.8 kbp), and *P. troglodytes*(AACZ03, 50.7 kbp). Table 1 provides details of the WGS statistics, particularly indicating the sequencing technology used, the coverage, and the N50 values.

### 2.2. The Iterative ML Algorithm: VgeneFinder

As described previously, V-genes may differ substantially between species, especially species distant in evolution, such as humans and reptiles (>300 M years of evolution). Search algorithms, such as BLAST, are extremely useful for obtaining genes with high homology but are not reliable when the sequences differ significantly. Thus, an algorithm that can infer sequences quite different from the known V-genes is needed. We implemented such an algorithm as a Python-based software package called VgeneFinder. The VgeneFinder software tool is an improvement over our previous method because it discovers V-genes with a probabilistic, alignment-free method, translating these sequences into numerical feature vectors and then applying a Random Forest classifier to determine whether the sequences are valid V-genes and to which loci they belong. Moreover, V-genes from distant taxa are incorporated into the systems knowledge base, allowing for more robust V-gene homology discovery.  Figure 1 illustrates the iterative steps of the VgeneFinder workflow.

**Figure 2:**
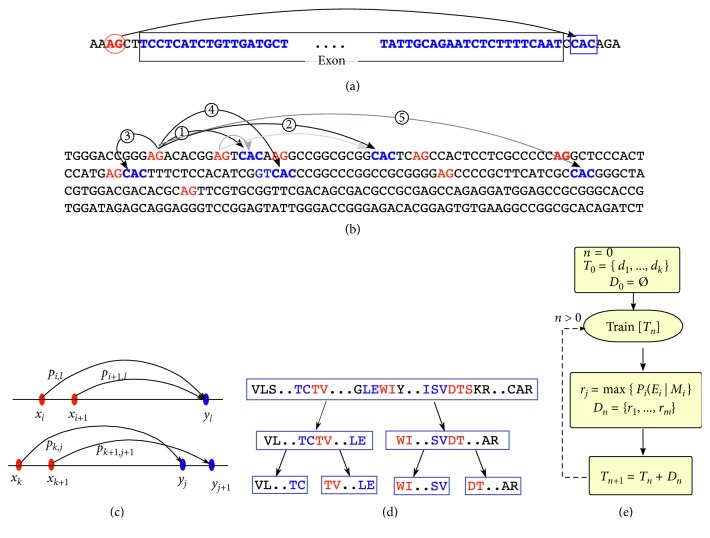
Process of obtaining candidate exon sequences. (a) The definition of an *in-frame* exon sequence between the -AG- start motif and the RSS canonical -CAC- motif. (b) Identification of all sequence possibilities between the AG-CAC motifs. (c) Examples of overlapping exon intervals; candidates are reduced with an interval tree, while best candidate V-genes are chosen by maximum probability. (d) Multiscale decomposition of a sequence stored as a recursive tree structure. (e) High-level flow diagram of steps of the iterative bootstrap training process: *n* is the iteration step, *T*_*n*_ is the set of V-exons used in training Random Forests (using >100 random trees and default parameters from the sklearn library) for each level, *D*_*o*_ are the new exons that have been discovered at step *n* and will be added to the *n*+1 iteration for training, and *E*_*j*_ and *M*_*i*_ represent exon intervals and training matrices, respectively, for which maximum likelihood criteria are applied.

The iterative training/prediction process stops when no more genes are further discovered. At this point, the algorithm has a high specificity for predicting homologous V-gene sequences with a low false-positive rate (<2%), but at the same time, sufficient probability for identifying V-genes far from canonical forms. In this way, the VgeneFinder algorithm uncovers the sequences identified by VgenExtractor as well as additional sequences, while at the same time, it classifies V-genes into their respective loci. As described previously, the VgeneFinder program identifies *in-frame* V-exon nucleotide sequences (Figure 2(a)) by searching WGS contigs (or scaffolds or chromosomes) from a Fasta file and finding exon boundaries defined by the AG start motif and terminated by three letters of the canonical *recombination signal sequence* (RSS)—the CAC motif, which is conserved throughout all jawed vertebrates and shown to be the same for all V-genes with only slight variations [19, 20] (i.e., the estimated variations of CAC are <1%).

In the VgeneFinder exon search algorithm, all AG-CAC sequence intervals are extracted and tested with necessary conditions for being viable exons; if valid, they are processed with Random Forest classifiers at each multiresolution levels.  Figure 2(b) illustrates how AG-CAC sequences are distributed and interspersed throughout a typical contig region. Only those sequences with size 275–330 bp (since V-genes are approximately 300 bp) are translated in-frame to amino acid sequences. A large fraction of these sequences are noncoding (i.e., containing stop codons in the reading frame) and can be immediately discarded. The remaining sequences are converted into a multiresolution feature vector in order to be used for predicting homology. Unique V-genes within a given nucleotide interval are predicted with a maximum likelihood criteria, resolving possible sequence overlaps (Figure 2(c)). Enumerating all AG-CAC intervals is computationally demanding for such large Fasta files. As such, we implemented an optimized algorithm based on an *interval tree* data structure (with the Banyan python library) that groups overlapping intervals defined by sequence start/stop contig positions.

**Figure 3:**
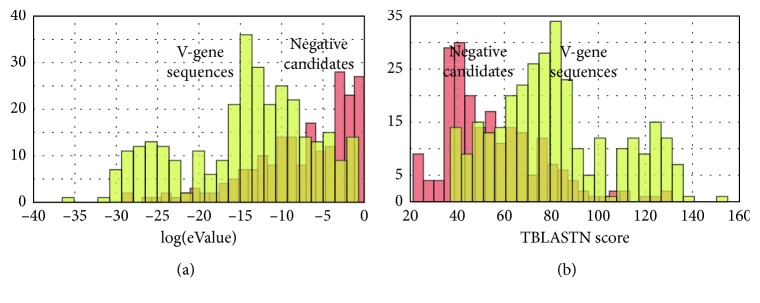
Comparison of TBLASTN hits compared to the V-gene sequences positively identified by VgeneFinder: (a) log(eValue) scores and (b) scores of TBLASTN for all candidates. The histogram negative candidates (red) are candidate sequences that VgeneFinder has discarded. The V-gene sequence histograms (yellow) are positively identified by VgeneFinder. The plots show that just based on TBLASTN homology, there would be no manner to determine positive and negative sequences; TBLASTN and similar homology methods are not effective for this task nor could they be used to automatically classify the exons into their respective loci.

An alternative method to the brute force enumeration of all intervals between the AG-CAC motifs is to use TBLASTN. While TBLASTN can act as a rough filter on potential V-genes, it is neither specific enough for discriminating V-genes and determining loci nor possible to detect the exon boundaries correctly. To illustrate, Figure 3 shows histograms of negative TBLAST hits (with a search using eValue = 1.0 and queries from consensus sequences from each IG and TR loci) against the *Macaca mulatta* WGS AANU01, together with hits that are positively identified as V-genes by the VgeneFinder algorithm. The plots demonstrate that a simple filter based on the eValue score is not adequate for identifying V-genes.

### 2.3. Multiresolution Feature Vectors

As known from protein homology studies, the numerical representation of the V-exon AA sequences is critical for classification. Three numerical feature vector transforms were studied: a simple vector based on the occurrence frequency of AA and pairs, a vector that uses physicochemical properties of AA, and a hybrid vector that uses the two methods at different scales of the MR sequence. The transformation vector based on AA occurrence frequency (the AA *pairs* method) is formed by concatenating two vectors: the histogram of each AA and the histogram of each pair of AA; with 20 AA, the resulting feature vector has a length of 440 integer values. For the transformation based on physicochemical properties, AAindex1 [21] is used together with a normalization procedure (PDT) [22] that captures the position of AA and neighboring correlations. The resulting vector is a normalized 500 element vector of floating point values. This PDT method can also capture longer correlations; however in practice, no improvements were seen in sequence discrimination results.

Because V-gene peptide sequences are relatively long (i.e., ∼90 AA) and such feature methods work best for shorter sequences, we developed a multiresolution (MR) sequence decomposition data structure, **S**_*ij*_, shown in Figure 2(d). In this structure, the original AA-deduced V-gene sequence is recursively subdivided into *j* sequences at hierarchical level *i* for which one of the transformation methods is applied. Such a structure allows for flexibility in applying transforms to the levels of the hierarchy. In the hybrid transform method, the AA pairs and PDT transforms are applied to different levels of this hierarchy. In another structure tested, the PDT was applied with different correlation lengths, *λ*, at each scale, so that longer correlations are captured on the highest layer in the hierarchy, while the bottom most layer captures the immediate neighboring correlations. When combined, the resulting data structure is a tree reminiscent of wavelet transformations, where each decomposition captures a different level of structure. Note that this method obviates the need for sequences to be aligned for classification.

Training and prediction of the Random Forest classifier is performed separately at each MR level (*i*, *j*). From the training matrices *M*_*ij*_ for the *i* multiresolution levels (*i*, *j*), binary probabilities are calculated for each locus, *L*_*k*_, from the ensemble classifier, so that {*p*_*ij*_(*L*_*k*_)=[*p*_0_, *p*_1_]|∀ (*i*, *j*) < *n*}, where [*p*_0_, *p*_1_] represents the background and signal probability, respectively. Therefore, the probability for a candidate AA sequence, *S*_c_, is expressed as the set *P*_s_={(*p*_*ij*_)_0_ ⋯ (*p*_*ij*_)_*K*_|∀ *L*_*k*_}. The loci with maximum probability are chosen by maximum likelihood: *L*_p_=argmax_*k*_(*P*_s_).

The probabilities at each multiresolution (MR) levels provide additional degrees of freedom for applying intuitive restriction criteria for selecting valid V-genes sequences. In particular, by demanding that the probabilities from each sequence segment are within a range *ϵ* of each other {|*p*_*i*+1,*j*_ − *p*_*i*,*k*_| < *ε*|∀ *i*, *j*, *k*}, it is equivalent to demanding that the sequences are homologous throughout. The value of *ϵ* in practice was chosen empirically to be ≈ 0.17, by observing many bootstrap training/prediction runs and comparing predictions with genes identified by VgenExtractor; the values of *ϵ*, together with the overall probability threshold, are free parameters and control the *homology bandwidth* for discovering sequences far from the *median* homology of the training set. Nonetheless, the choice of these parameter values does not significantly affect the results of the most of the machine learning predictions. A further important condition that guarantees that the exon boundaries correspond to functional V-genes is imposed on the subsequences at the extreme ends of the AA translated exon, corresponding to the left-most (*L*) and right-most (*R*) sequences of the lowest MR level (*n*) or *S*_*nL*_ and *S*_*nR*_, respectively. This condition corresponds to {*p*_*i*^*∗*^_(*L*); *p*_*i*^*∗*^_(*R*)} > *τ*, where *i* ^*∗*^ is the maximum subdivision, *L* and *R* refer to the left-most and right-most subsequences, and *τ* is the threshold (in practice *τ* ≈ 0.7).

### 2.4. Online Iterative Learning

Given the numerical representation of the AA sequence, supervised machine learning is used to train a Random Forest ensemble classifier [23] from a small initial set of known V-genes obtained from *Homo sapiens* and *Mus musculus* obtained from the IMGT [24] and Ensembl [25]. Binary training, consisting of positive (functional V-genes) and background (random) sequences, is performed for each locus and at each multiresolution level. The background sequences are selected randomly with a signal ratio of 3 : 1 and shuffled for each multiresolution level training matrix, *M*_*ij*_. From the initial training matrices, V-gene prediction is carried out with 14 WGS primate datasets; positively selected V-genes are incorporated into the subsequent round of training. This *online* (i.e., incremental and iterative addition of new training data) supervised learning procedure is repeated until no additional new genes are discovered upon further iterations.  Figure 2(e) shows the general steps of this procedure in a flow diagram.

### 2.5. Practical Implementation

VgeneFinder is a multithreaded application (using a MapReduce design pattern) that concurrently divides large WGS contigs into smaller overlapping chunks for V-exon search and processing (in practice, the chunk size is 20 kbp, with an overlap of 1 kbp). In each chunk, a map processing phase identifies candidate exon intervals, which are then combined in a reduction phase, thereby removing possible duplicates from the overlaps. For each candidate, the MR predictions are made for each V-gene isotype. As mentioned previously, WGS Fasta files are approximately  3G consisting of ≈3 × 10^5^ contigs with N50 >15 kbp, but average contig sizes are ≈100–200 kbp. The average processing time for the WGS files of primates is approximately 2.5 minutes/contig on a modest desktop Linux PC (i.e., Intel Core i5-2400 CPU 3.10 GHz 4-core i5 Intel processor, running the Linux kernel 3.2).

## 3. Results

The bootstrap learning algorithm iteratively improves the ensemble class probabilities for predicting each locus. The process was applied to 14 WGS primate datasets, independently testing each of the feature vector transforms. Binary training with a Random Forest classifier was carried out for each V-gene isotype, *k* (resulting in matrices *M*_*ij*_(*k*)), using the sklearn [26] library with 500 trees and a signal/background ratio of 3 : 1 (as described in Methods). For all candidate exon sequences at each iteration *t*, sequences were converted to an MR structure **S**_*ij*_ and binary predictions made for each locus with *M*_*ij*_(*k*, *t*). The predicted class probabilities, *p*_*ij*_(*k*), obtained from each MR level were combined into a single score, which served as the basis for selecting sequences with respect to an adaptive threshold. The MR score, *τ*=*N* − 1/*N*∑_*k*_∑_*m*_(1 − exp(|*p*_00_ − *p*_*km*_|^2^/*σ*)), is degraded if the probability at different MR levels (*p*_*km*_) deviates significantly from the probability *p*_00_ of the zeroth-level MR sequence.

**Figure 4:**
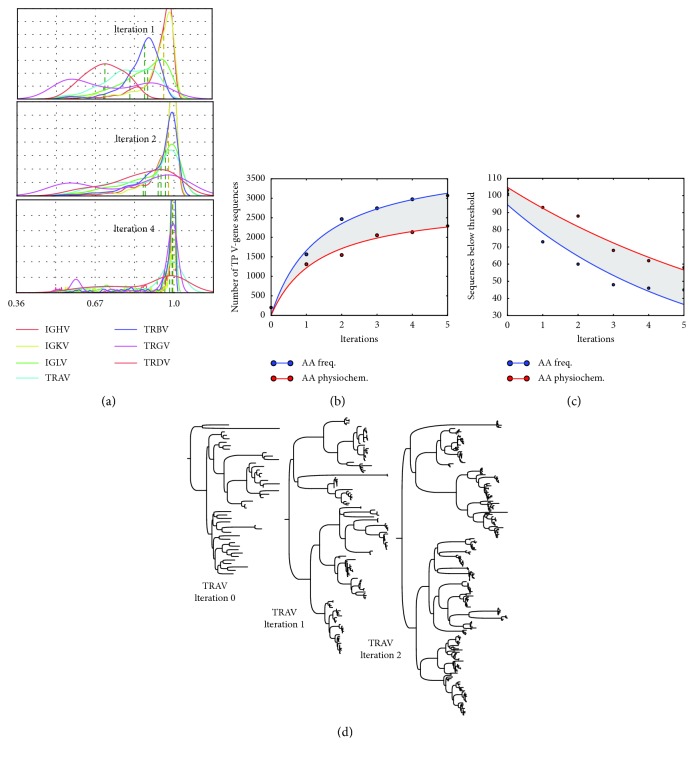
(a) Density distributions of the iterative learning algorithm of VgeneFinder for successive iterations using 14 WGS primate datasets. (b) Number of total sequences as a function of iterations for two different feature vector transforms; the AA frequency transform considers consecutive pairs of amino acids, while the AA physicochemical is a method that forms a feature vector using physical properties depending on the position of amino acids. (c) The number of sequences that are below the prediction threshold as a function of iteration, indicating that exons which are quite distant from the initial training set (but nonetheless viable V-genes), are gradually included as the iterative process evolves. (d) Example of TRAV multispecies tree for starting set (with *H. sapiens*) and 2 iterations (see more detailed view in Figure 5(c)).

The distributions of predicted sequences based on their MR scores are visualized with a histogram and fit to a kernel density estimation (KDE).  Figure 4(a) shows KDE probability distribution results from successive learning/prediction iterations of the bootstrap process corresponding to the AA-pair transform (Section 2); the KDE results for the other feature transforms behave similarly. As can be seen, in the first iteration step, the KDE distributions are broad and have low mean probabilities. Upon successive iterations, the mean probability of predicted sequences move towards higher values and the KDE distributions of all loci are more sharply peaked, indicating that predictions of V-genes have a high specificity and (with constant area) most sequences are under peak of the distribution.

**Figure 5:**
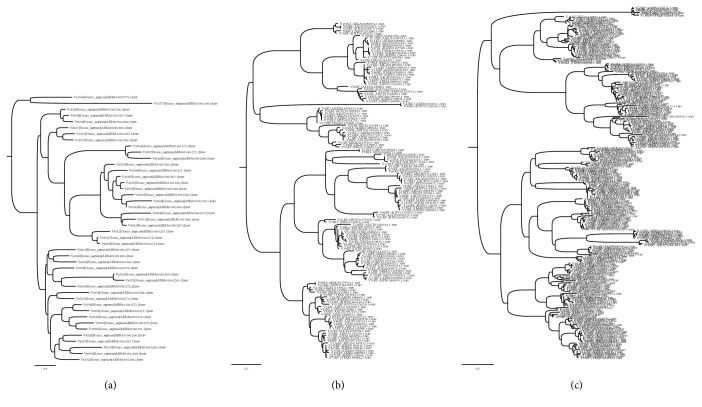
Two iterations of the TRAV tree using the bootstrap method and showing the branch labels of each taxon. With each iteration, more branches are discovered from the 14 WGS primate data and included in subsequent training. The VgeneFinder algorithm classifies the V-genes according to their loci. Here only the V-genes pertaining to the TRAV locus are shown.

Figure 4(b) (top) shows results of the total number of *true-positive* (TP) V-genes as a function of iteration *t*, comparing two-feature vector transforms: AA pairs and PDT. Figure 4(b) (bottom) shows the number of sequences discarded at each iteration whose probability was below threshold.  Figure 4(c) shows the phylogenetic tree of the TRAV loci at each iteration step. From these plots, it is clear that the best method is the AA pairs methods for forming the feature vector (i.e., AA pairs are based on the occurrence frequency of amino acids and pairs of consecutive amino acids).  Figure 5 shows a more detailed view of the TRAV locus in the iterative discovery of V-genes.

### 3.1. Validation of VgeneFinder with Known Sequences

To validate VgeneFinder, we compared the genes found by this software with the available V-gene annotations of the IMGT and with our previous software, VgenExtractor. The sequences annotated by the IMGT (and deposited in the Ensembl database) were obtained through laborious multiple experimental methods. As such, these sequence annotations are accepted by the scientific community as gold standards.

As described previously, other standard gene finding software is not valid for discovering V-genes because the V-exon boundaries do not follow canonical rules. As such, the automatic V-exon annotations provided in new genome projects that use classic gene finding software have significant deficiencies in reporting the actual number of V-exons. Through validation with the known IMGT sequences, our software accurately automates V-exon annotation and can be used to identify V-genes newly available genomes.

### 3.2. Multispecies Trees and Comparison with VgenExtractor

**Figure 6:**
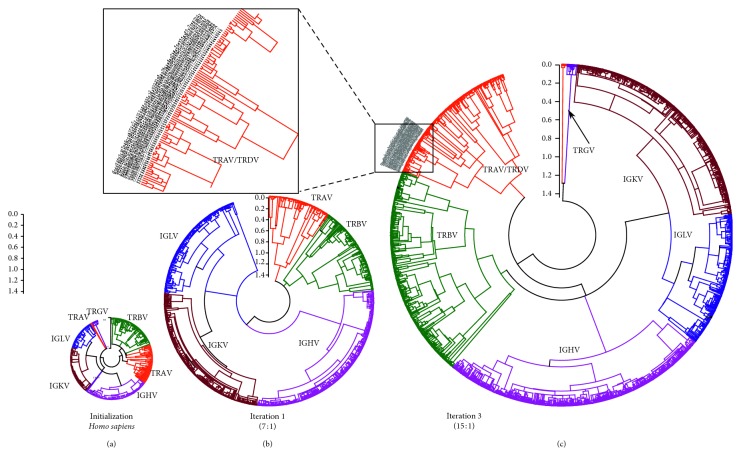
Phylogenetic trees of the amino acid sequences of V-exons for each iteration step. (a) Positively identified V-exon sequences are classified into their respective locus; the clearly delineated clades (i.e., IGHV, IGLV, IGKV, TRAV/D, TRBV, and TRGV) show that this classification is correct. The V-exon sequences were aligned with Clustal omega [27]. For constructing the phylogenetic trees, a maximum likelihood algorithm with the WAG matrix and 500 bootstrap replicates were realized for validation. Rooting was performed at the midpoint, and linearization provided by Mega [29] was applied to improve the visualization of the trees. In the initial iteration (b), only known V-exon sequences from humans and mouse were used in the training set. From this training, predictions were made by processing 14 WGS of primates; the discovered sequences from these primates were used to retrain Random Forests, thereby refining the possibility of including V-genes that are more distant in homology. In the third iteration (c), the program VgeneFinder uncovered 15 times more sequences than from the start of the iteration. For illustration, sequences from a small section of the TRAV are amplified (inset). More details of the branch distances can be found in Supplementary Materials.

The predicted sequences obtained by applying the iterative algorithm to the 14 WGS primate datasets were used to construct a multispecies V-gene tree. In particular, phylogenetic trees were constructed using clustalO [27] alignment and FastTree [28] with the WAG matrix and 500 bootstraps to produce newick files. For the tree construction, we used a maximum likelihood algorithm and the LG matrix. Finally, we used the MEGA (ver. 5) [29] (https://www.megasoftware.net/) and FigTree (http://tree.bio.ed.ac.uk/software/figtree/) to produce tree graphics.  Figure 6 shows the resulting trees at different iteration steps, starting with the initial training set (consisting of *H. sapiens* and *M. musculus*). These results provide a separate test of the VgeneFinder loci classification since the predicted loci form the well-defined clades as expected.

**Figure 7:**
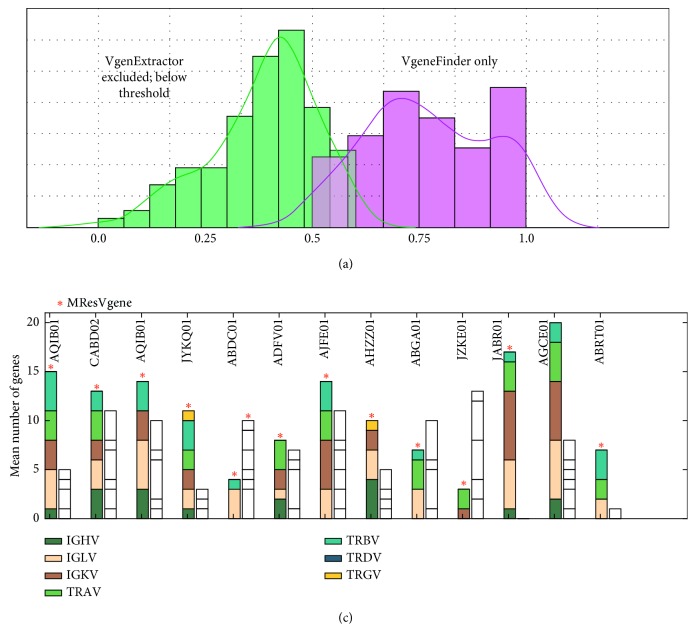
Prediction results comparing VgeneFinder and VgenExtractor. (a) The class probability of sequences predicted by VgeneFinder (right curve) that were not predicted by VgenExtractor and those not accepted by VgeneFinder (left curve) having class probabilities <0.5. (b) Sequence alignment comparison of two typical TRAV sequences and sequences not detected by VgenExtractor but predicted by VgeneFinder.

All VgenExtractor sequences were processed with AA-pair transform feature vector and scores calculated with the VgeneFinder predictor. All sequences of VgenExtractor are detected; however, many are discarded because of low MR classification scores. Phylogenetic comparisons are shown in Supplementary Materials (available here).  Figure 7(a) summarizes the results for sequences that did not agree (sequences predicted by VgenExtractor but discarded by VgeneFinder and those found by VgeneFinder but not found by VgenExtractor). Low scores for sequences indicate that they are far from the homology in the training set, not necessarily that they are nonfunctional V-genes. Moreover, the VgeneFinder score provides a homology metric, indicating which V-gene sequences can be considered with high confidence. Such information was not available previously with the VgenExtractor tool.

### 3.3. Validation from the Prediction of V-genes from Known Genes in *Macaca mulatta*

We validated the algorithm by studying the nonhuman primate, rhesus macaque (*Macaca mulatta*), whose genome is complete in the IG/TCR locus. The rhesus macaque (*Macaca mulatta*) is one of the most studied primates (apart from *H. sapiens*) because it is an ideal laboratory surrogate model for human disease and treatment. As such, the genome of the macaque is known in great detail, sharing approximately 93% of genes with *H. sapiens*, with complete chromosome reconstruction (21 pairs) and 3097.37 Mb. Gene annotation WGS pipelines have taken advantage of the alignment with the human genome, uncovering a large number of coding/noncoding genes. Nonetheless, the V-gene repertoire in this species has not been fully annotated yet.

In the training phase, the 14 WGS primates with VgeneFinder, and the genome of *M. Mulatta* was excluded so that prediction validation could be carried out and compared with V-gene annotations. IG and TCR V-genes annotations are available from the Ensembl repository [25] as a WGS assembly (*MMul ver. 1.0*) that maps to chromosome and/or scaffolds. For each gene in Ensembl, the corresponding protein transcript was downloaded from the UniProt database. The protein transcript sequences were saved in Fasta format for direct comparison with the sequences obtained with VgeneFinder and VgenExtractor from the nucleotide chromosome or scaffold segments.

All annotated sequences were used in the validation, except for three sequences (TRAV12-1, TRAV12-2, and TRBV4-1) which are only partial transcripts, not having a minimum length. Nonetheless, the Ensembl annotations are far from complete. At present, five IGHV sequences are located nonchromosomal scaffolds of the assembly, eight IGLV are in Chr10, four IGKV in Chr13, 16 TRAV are found in Chr7, and nine TRBV are in Chr3. No TRGV sequences are found, and there is one delta chain, TRDV, found in a scaffold region.

**Figure 8:**
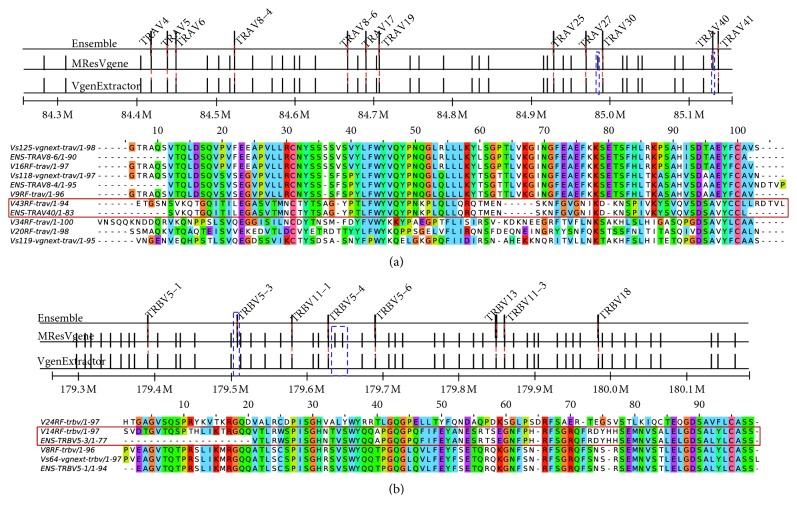
V-genes in *Macaca mulatta*. Comparison of V-genes obtained from VgeneFinder and VgenExtractor for TRAV and TRBV against the Ensembl annotations. The gene annotations in *M. mulatta* are limited as described in the text. This comparison shows that our software tools correctly identify all the known annotated genes as well as identify the rest of the V-gene repertoire. The comparison between VgeneFinder and VgenExtractor shows that VgeneFinder is able to uncover sequences which are not canonical (as seen in the alignments).

A summary of the comparison results between the VgeneFinder algorithm and VgenExtractor is shown in Table 2. VgeneFinder detects nearly 100% of the Ensembl annotated genes, except for IGLV1-51, which is only a partial sequence and whose functionality is questionable (Supplementary Materials).  Figure 8(a) shows a detailed comparison of the two methods with Ensembl TRAV and TRBV loci, in segments of Chr 7 and Ch3, respectively. The discrepancy between VgeneFinder and VgenExtractor for detecting Ensembl sequences can be understood in the sequence alignments (Figure 8(b)); sequences (ENS-TRAV40/1-83 and ENS-TRBV5-3/1-77) were detected by VgeneFinder fact but not VgenExtractor because they lack conserved motifs (i.e., ENS-TRAV40 lacks a cysteine between locations 15-28, and ENS-TRBV5-3 lacks a common Y^*∗*^ motif in the last 15 AA). Detailed IG comparisons and phylogenetic trees are shown in Supplementary Materials.

## 4. Discussion and Conclusions

The evolution of the vast majority of V-genes found throughout jawed vertebrate orders has progressed with a high degree of conservation at particular positions along the germline sequence. Structural or functional requirements of the resulting antigen-binding V domains may be responsible for such canonical motifs. Previous methods have exploited this structure but are unable to identify V-genes having less common motifs or extending the algorithm to more distant species such as bony fish, with additional IG and TCR isotypes. The iterative learning algorithms of VgeneFinder provides an alignment-free probabilistic method for obtaining V-genes with high specificity for homologous genes but can be used to gradually expand the original set to evolutionary distant taxa. The probabilistic scores of the classifier provide an alignment-free homology distance metric which can serve as a confidence score for V-gene sequences. This quantitative metric can be used to rule out sequences when no other information is available, such as gene expression transcripts.

Apart from the iterative ensemble learning processes itself, there are two features that contribute to the success of this algorithm. The first is the multiresolution decomposition of the deduced amino acid sequences, and the other is the choice of the feature vector transformation. Because these V-genes are relatively long homologous germline exon sequences (≈300 bp), a single transformation does not provide enough local information of the sequence to properly distinguish homology; the prediction probabilities from multiple levels of the sequence probe the sequence at multiple scales. Finally, while the iterative online learning method was applied here to V-genes, it is general and could be used more broadly for homologous gene discovery in situations where the exon structure is well understood.

## Figures and Tables

**Table 1:** WGS data for the 16 primates included in this study.

Species	WGS and bio project no.	PubMed citation	Sequencing coverage	Contig N50
Lemuriformes				
*D. madagascariensis*	AGTM00000000.1 PRJNA74997	22155688	Illumina GA IIx 38×	3,653
*O. garnettii*	AAQR00000000.3 PRJNA16955	NP/DS	Illumina GA IIx 137×	27,100
*M. murinus*	AAHY00000000.1 PRJNA11785	12040188	Celera	21,690

Tarsiiformes				
*T. syrichta*	ABRT000000000.1 PRJNA20339	NP/DS	Sanger 2.07×	38,165

New World monkeys				
*C. jacchus*	ACFV00000000.1 PRJNA20401	NP/DS	ABI 3730 6.6×	29,273
*S. boliviensis*	AGCE00000000.1 PRJNA67945	NP/DS	Illumina HiSeq 80×	38,823

Old World monkeys				
*M. mulatta*	AANU00000000.1 PRJNA12537	17431167	Sanger	25,707
*M. fascicularis*	CAEC00000000.1 PRJEA48347	21862625	454-FLXr SOLiD	8,925
*C. sabaeus*	AQIB00000000.1 PRJNA168621	NP/DS	454 titanium; Illumina HiSeq; ABI	90,449
*P. anubis*	AHZZ00000000.1 PRJNA54005	NP/DS	Sanger: 2.5× 454: 4.5× Illumina: 85×	40,262

Hominids				
*N. leucogenys*	ADFV00000000.1 PRJNA13975	NP/DS	Sanger 5.6×	35,148
*P. abelii*	ABGA00000000.1 PRJNA20869	21270892	Sanger 6×	15,648
*G. gorilla*	CABD00000000.2 PRJNA169344		Sanger	
*P. paniscus*	AJFE00000000.1 PRJNA49285	22722832	454 26×	66,775
*P. troglodytes*	AACZ00000000.3 PRJNA13184	16136131 16136134	Sanger 6×	50,656
*H. sapiens*	ABBA00000000.1 PRJNA19621	17803354	Sanger	108,431

NP: no publication; DS: direct submission.

**Table 2:** Prediction comparisons with the annotated genes of *M. mulatta* obtained from the Ensembl (ENS) repositories. Predictions results of the total and true positives (TPs) against ENS of VgeneFinder (MRV) and VgenExtractor (VE) are shown.

Locus	Gen. loc.	ENS	(MRV and VE)	TP (MRV and VE)
TRAV	Chr7	12	46/43	12/11
TRBV	Chr3	8	56/53	8/7
IGHV	Scaff	3	32/31	3/3
IGKV	Chr13	3	35/31	3/3
IGLV	Chr10	8	41/36	7/6

## Data Availability

All genome WGS data used in this study were obtained from the public repository at NCBI (http://www.ncbi.nlm.nih.gov) with the detailed accession numbers provided in the manuscript. The genes extracted by our software described in this study have been deposited online in the VgeneRepertoire.org repository (see description at https://doi.org/10.1101/002139).

## Supplementary Materials

Supplementary Materials supp_materialsV2.pdf: this is an updated document, which includes new figures requested by the reviewer. sequences_alignments.zip: these are genome sequence files that are relevant for reproducing some of the results.
