# Supplementary material for: Iterative Variable Gene Discovery from Whole Genome Sequencing with a Bootstrapped Multiresolution Algorithm
Source: Comput Math Methods Med. 2019 Feb 11;2019:3780245. doi: 10.1155/2019/3780245 (PMC6388353; doi:10.1155/2019/3780245)
Supplement: Supplementary Materials — supp_materialsV2.pdf: this is an updated document, which includes new figures requested by the reviewer. sequences_alignments.zip: these are genome sequence files that are relevant for reproducing some of the results. [file 3780245.f1.zip › 3780245.f1/supp_materialV2.pdf]

Iterative V gene discovery from Whole Genome Sequencing with a bootstrapped Multiresolution Algorithm

David N. Olivieri and Francisco Gambón-Deza

1. Detail Branch distances for bootstrap iterations

The following are molecular phylogenetic trees showing the branch distances. These plots accompany the plots of Figure X in the manuscript. Notice that here, the clades are not colored as in the manuscript. These figures can be reproduced with the Fasta and nwk files supplied; using Mega5, select “root at midpoint”, “circular tree style” and “linearized tree”.

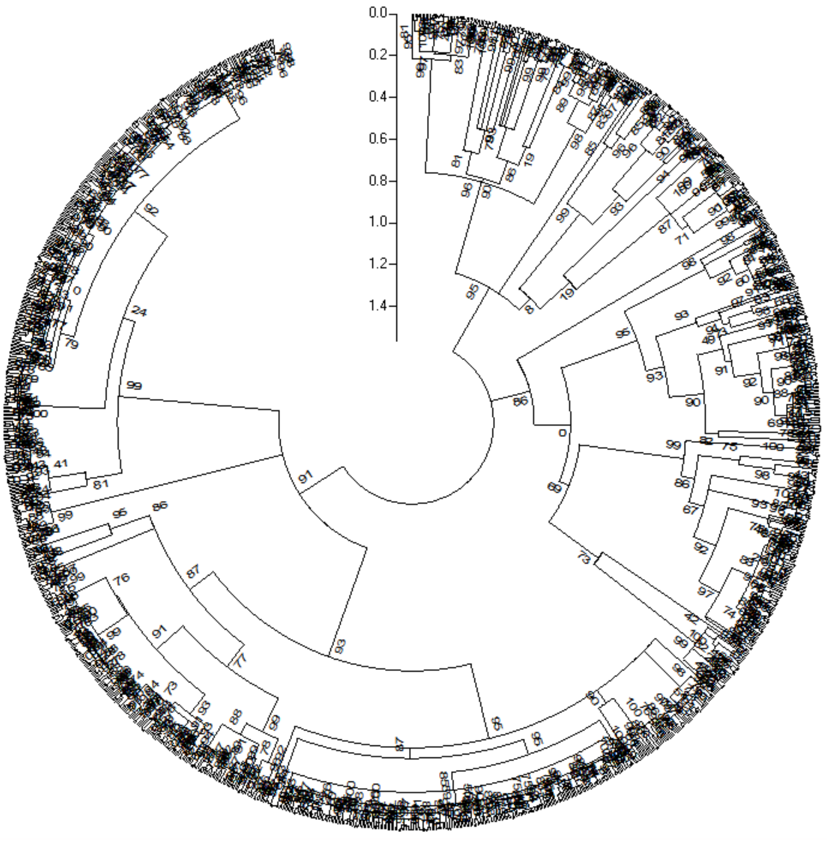

iteration-0

Figure S1: Phylogenetic tree of the initial set of V exons showing branch distances. Iteration-0 of the iterative process.

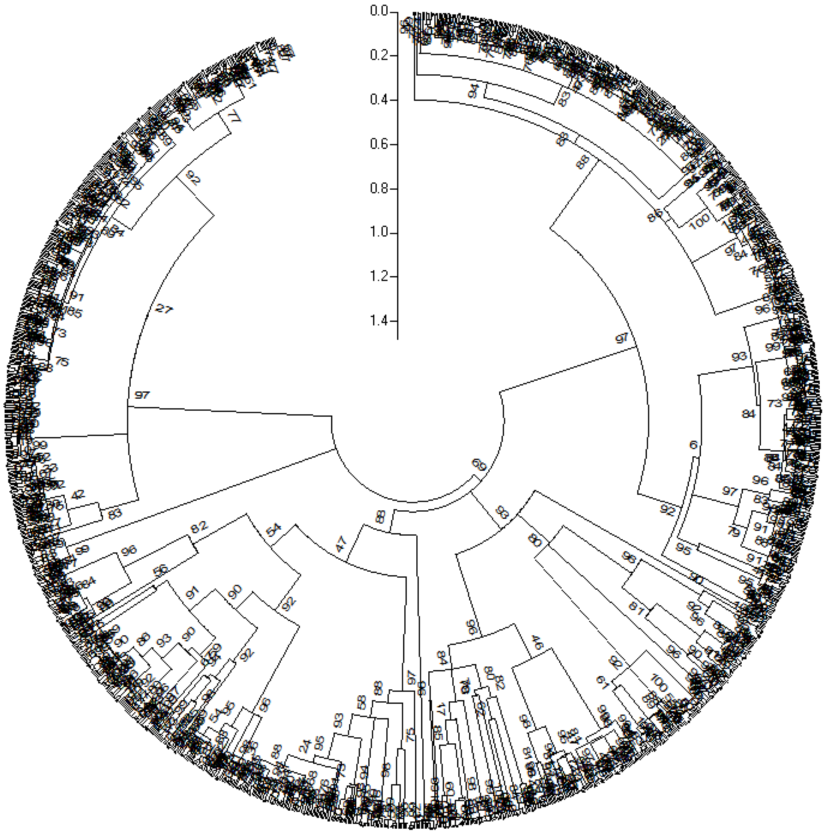

iteration-1

Figure S2: Phylogenetic tree of the initial set of V exons showing branch distances. Iteration-1 of the iterative process.

2. Comparison between MResVegene and VgenExtractor

2.1. Loci comparisons for Chlorocebus sabaeus

Representative comparisons of the V-genes discovered with MResVegene and VgenExtractor are shown in the phylogenetic trees for all the loci (Figures S4 – S9) for the Old World Monkey, *Chlorocebus sabaeus* (WGS accession AQIB01, N50=90k). Because these are on contigs of the WGS, a comparison was made by showing phylogenetic trees; common branches indicate that the sequences are equal. Similar trees are available in accompanying nwk and fasta files. The branch labels indicate consist of the Vgene name (the names with `RF` are from MResVgene) and the numbers indicate the total MRscore (see methods, highest value is 3.0), as well as the score in each multiresolution level. Scores assigned to VgenExtractor sequences were obtained by processing with the MResVgene predictor.

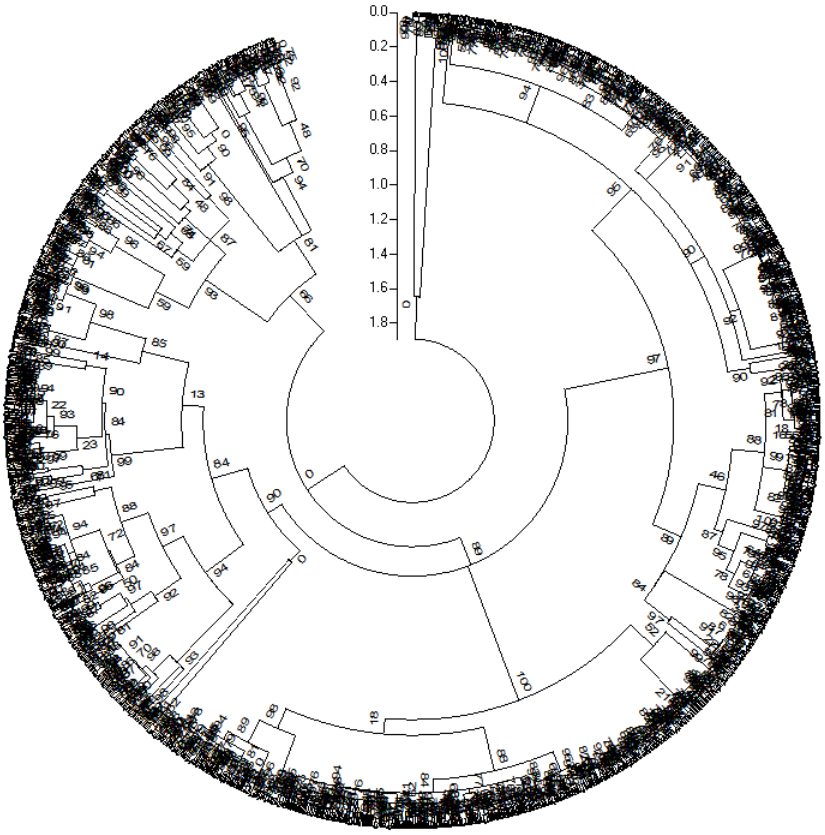

iteration-3

Figure S3: Phylogenetic tree of the initial set of V exons showing branch distances. Iteration-3 of the iterative process.

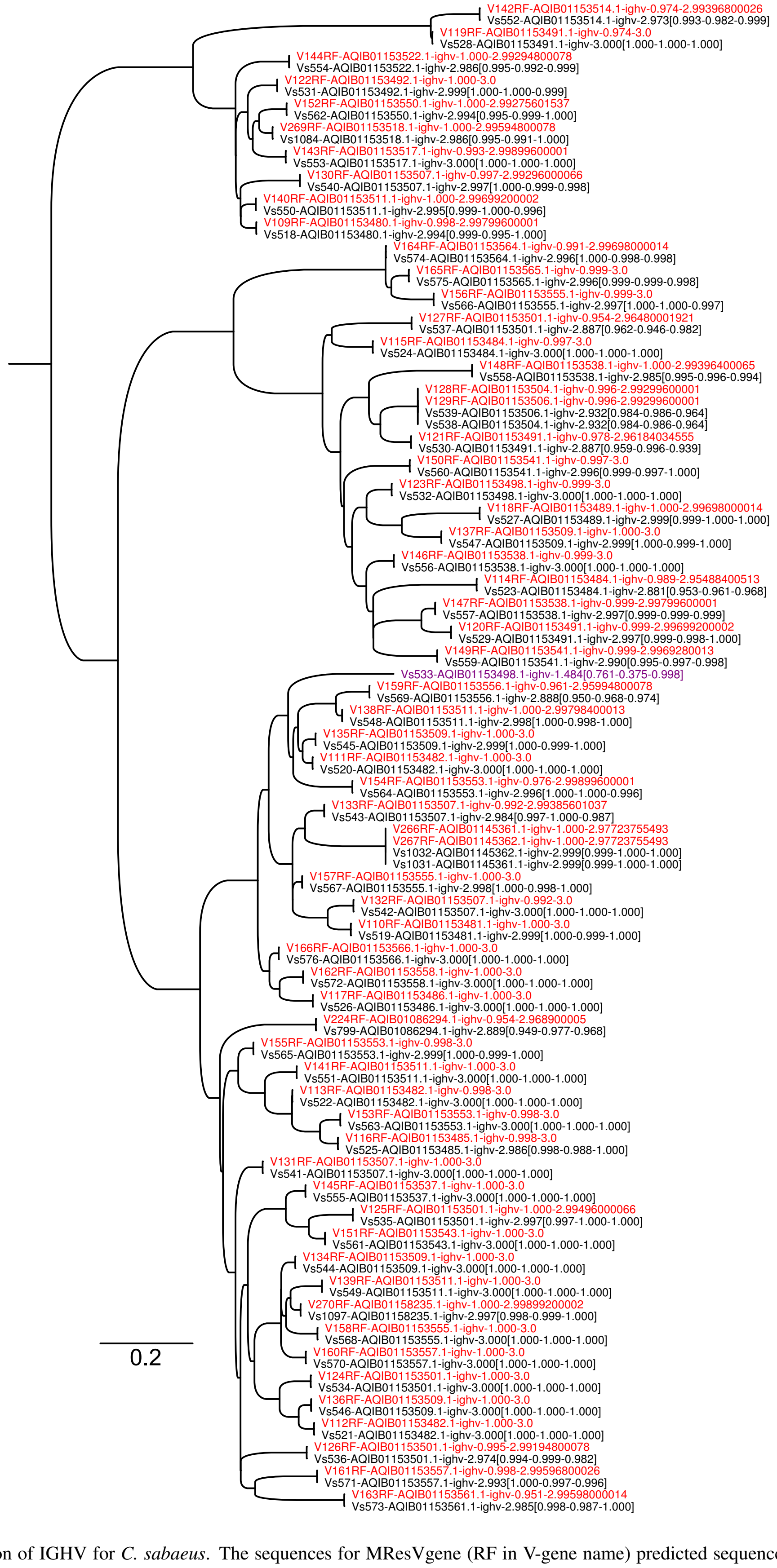

Figure S4: Comparison of IGHV for *C. sabaeus*. The sequences for MResVgene (RF in V-gene name) predicted sequences that are common to VgenExtractor are indicated in red; only predicted by MResVgene are in blue, and only predicted by VgenExtractor (purple).

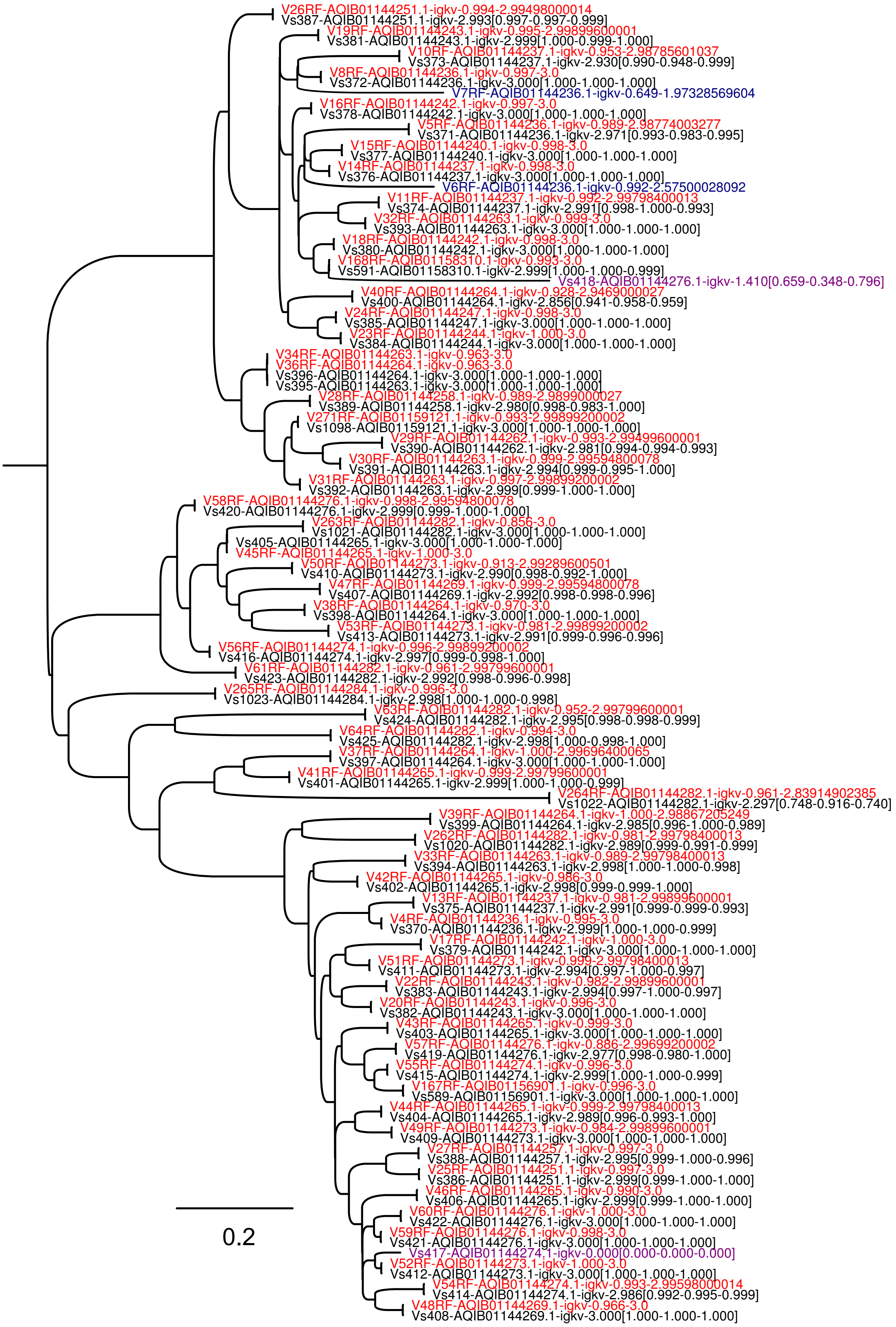

Figure S5: Comparison of IGKV for *C. sabaeus*. The sequences for MResVgene predicted sequences that are common to VgenExtractor are indicated in red; only predicted by MResVgene are in blue, and only predicted by VgenExtractor (purple).

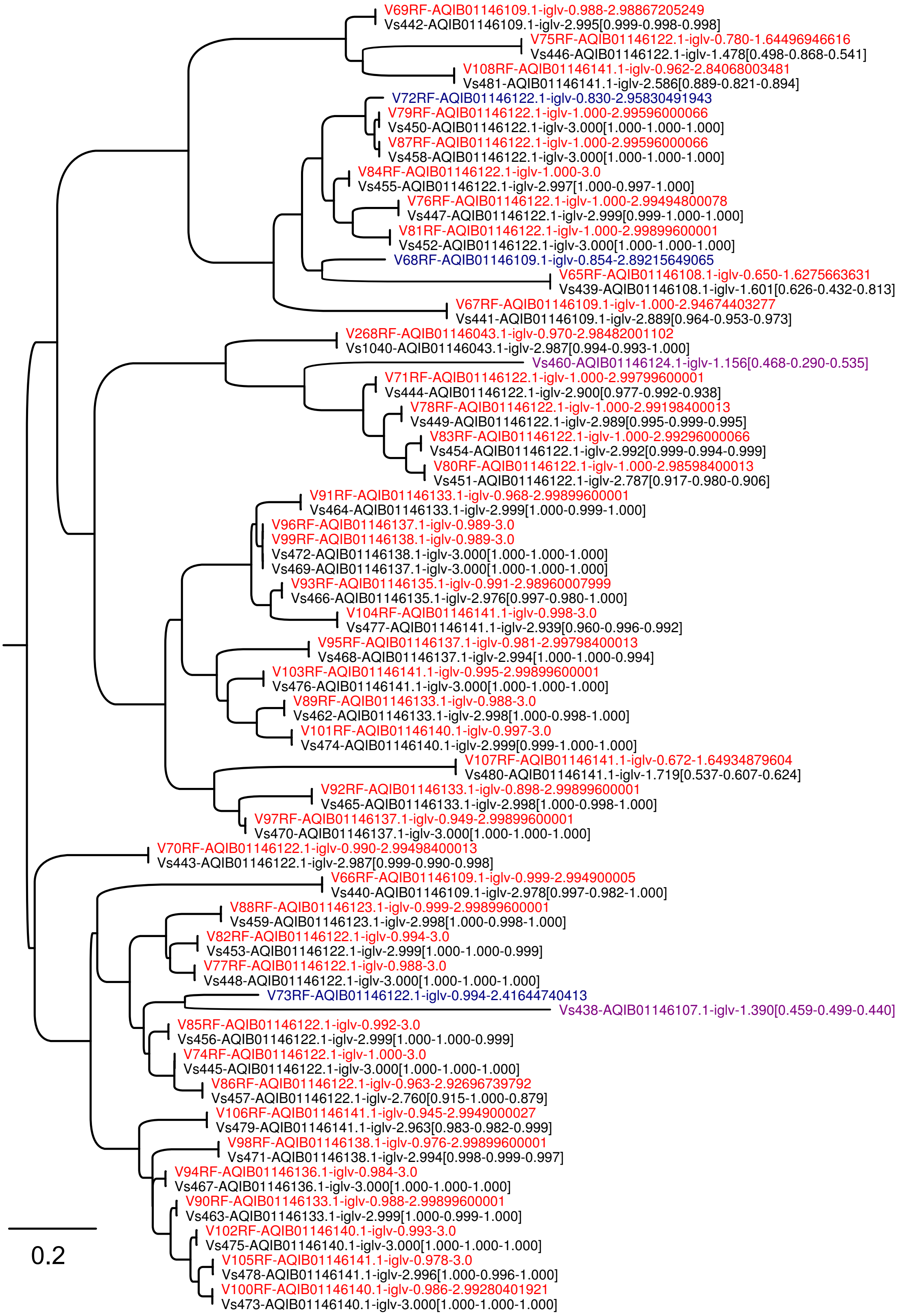

Figure S6: Comparison of IGLV for *C. sabaeus*. The sequences for MResVgene predicted sequences that are common to VgenExtractor are indicated in red; only predicted by MResVgene are in blue, and only predicted by VgenExtractor (purple).

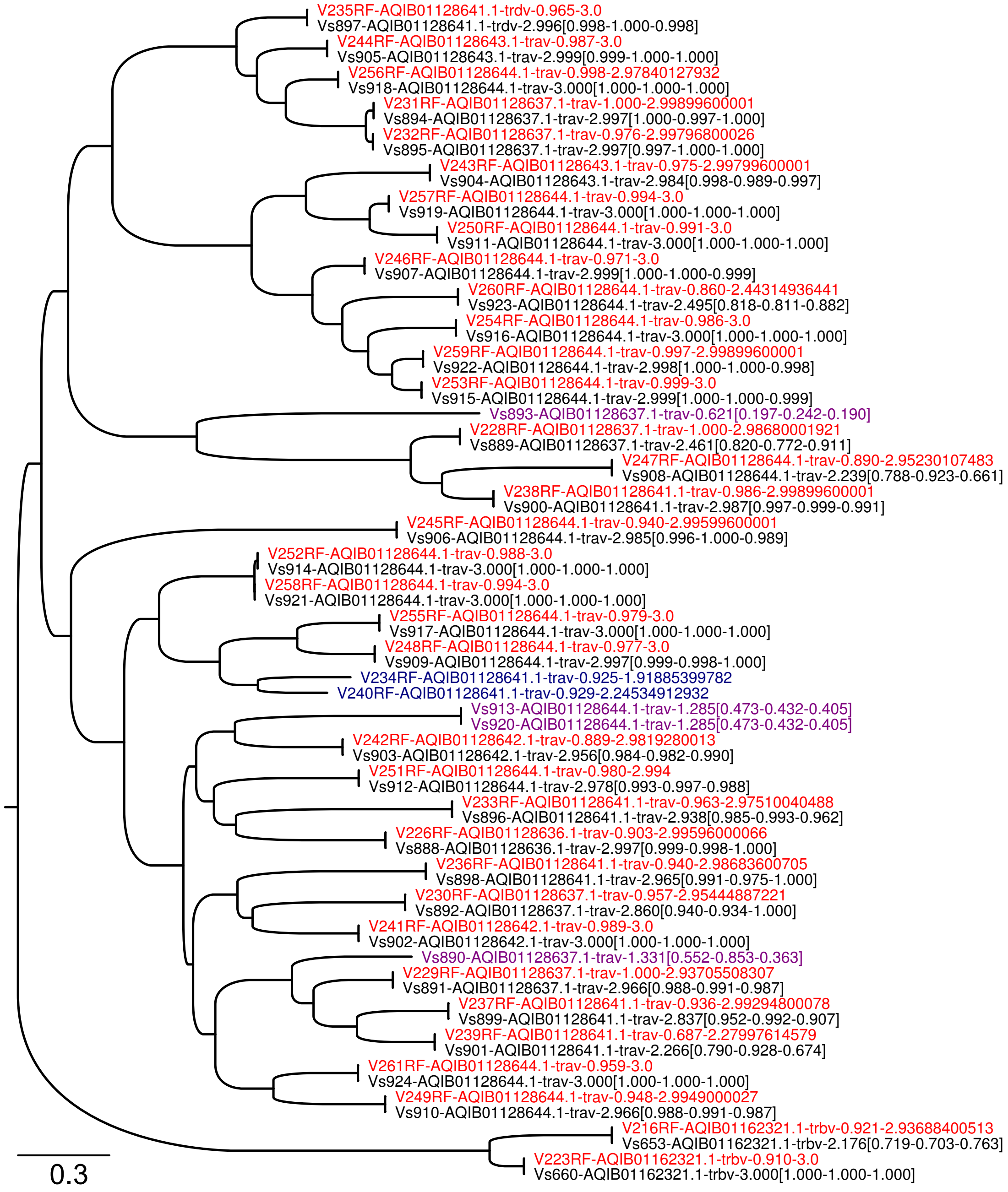

Figure S7: Comparison of TRAV for *C. sabaeus*. The sequences for MResVgene predicted sequences that are common to VgenExtractor are indicated in red; only predicted by MResVgene are in blue, and only predicted by VgenExtractor (purple).

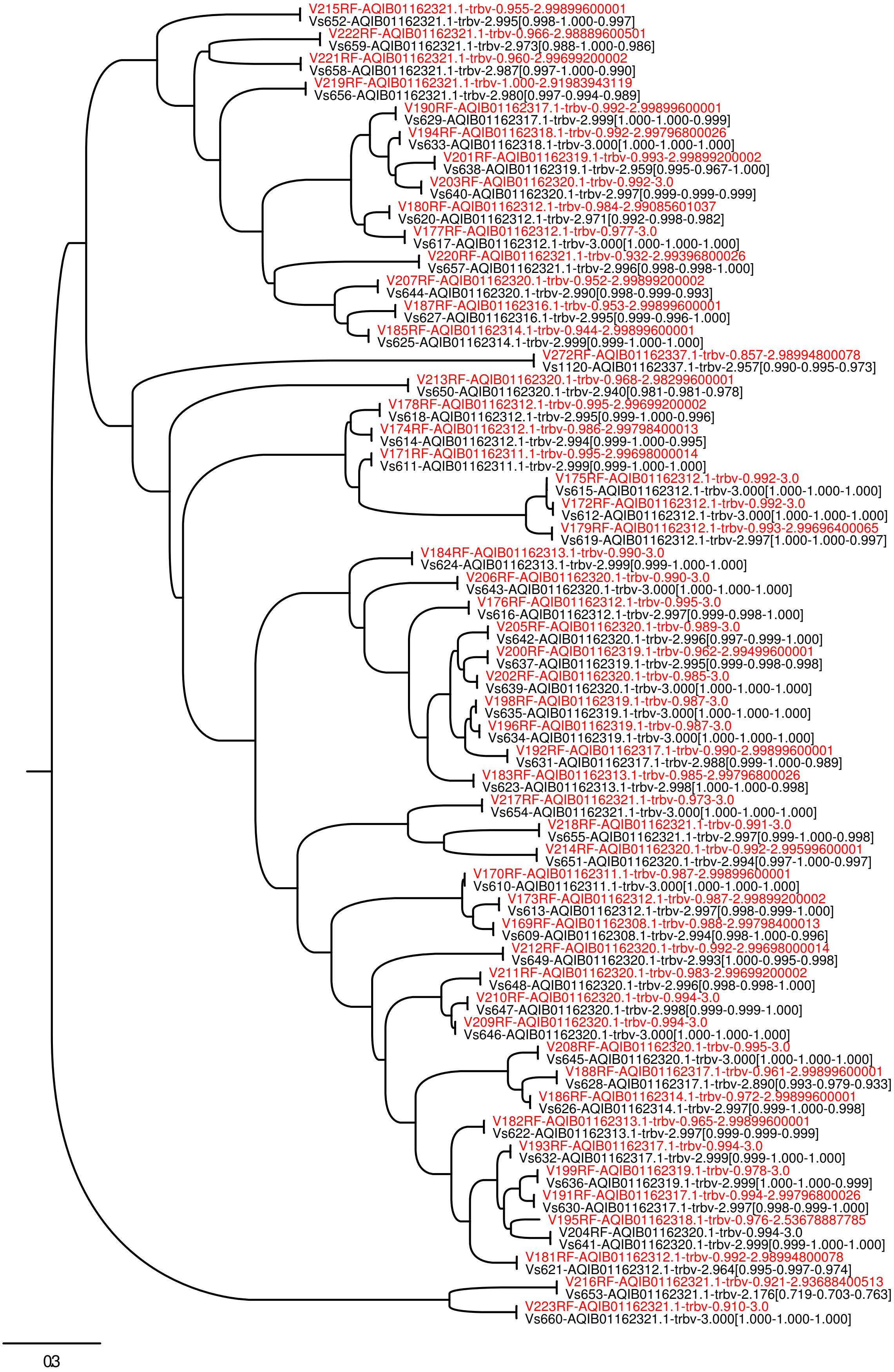

Figure S8: Comparison of TRBV for *C. sabaeus*. The sequences for MResVgene predicted sequences that are common to VgenExtractor are indicated in red; only predicted by MResVgene are in blue, and only predicted by VgenExtractor (purple).

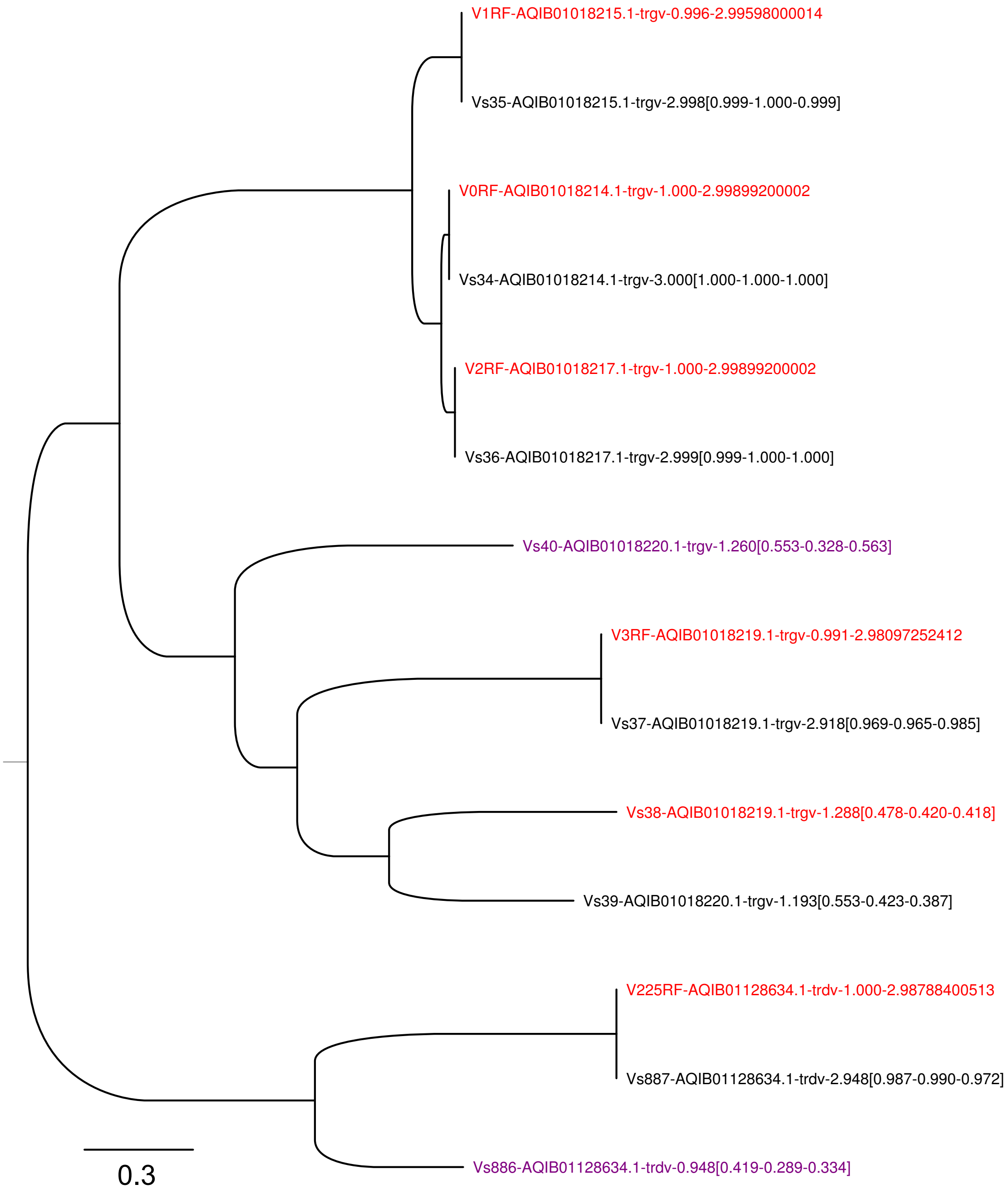

Figure S9: Comparison of TRGV for *C. sabaeus*. The sequences for MResVgene predicted sequences that are common to VgenExtractor are indicated in red; only predicted by MResVgene are in blue, and only predicted by VgenExtractor (purple).

2.2. Non-overlapping sequences between MResVegene and VgenExtractor

Figures S10 and S11 show the phylogenetic and alignment, respectively, of sequences discovered by MResVgene but not by VgenExtractor. All the sequences have high probability (MRscore) for being V-genes. The sequences also form well defined clades in S10.

Figures S12 and **??** show the phylogenetic and alignment, respectively, of sequences discovered by VgenExtractor but not by MResVgene All the sequences have low probability (MRscore) for being V-genes. While the low MRscore does not necessarily indicate that these sequences are non-functional V-genes (although some are), it does provide a metric for their distance from common homology.

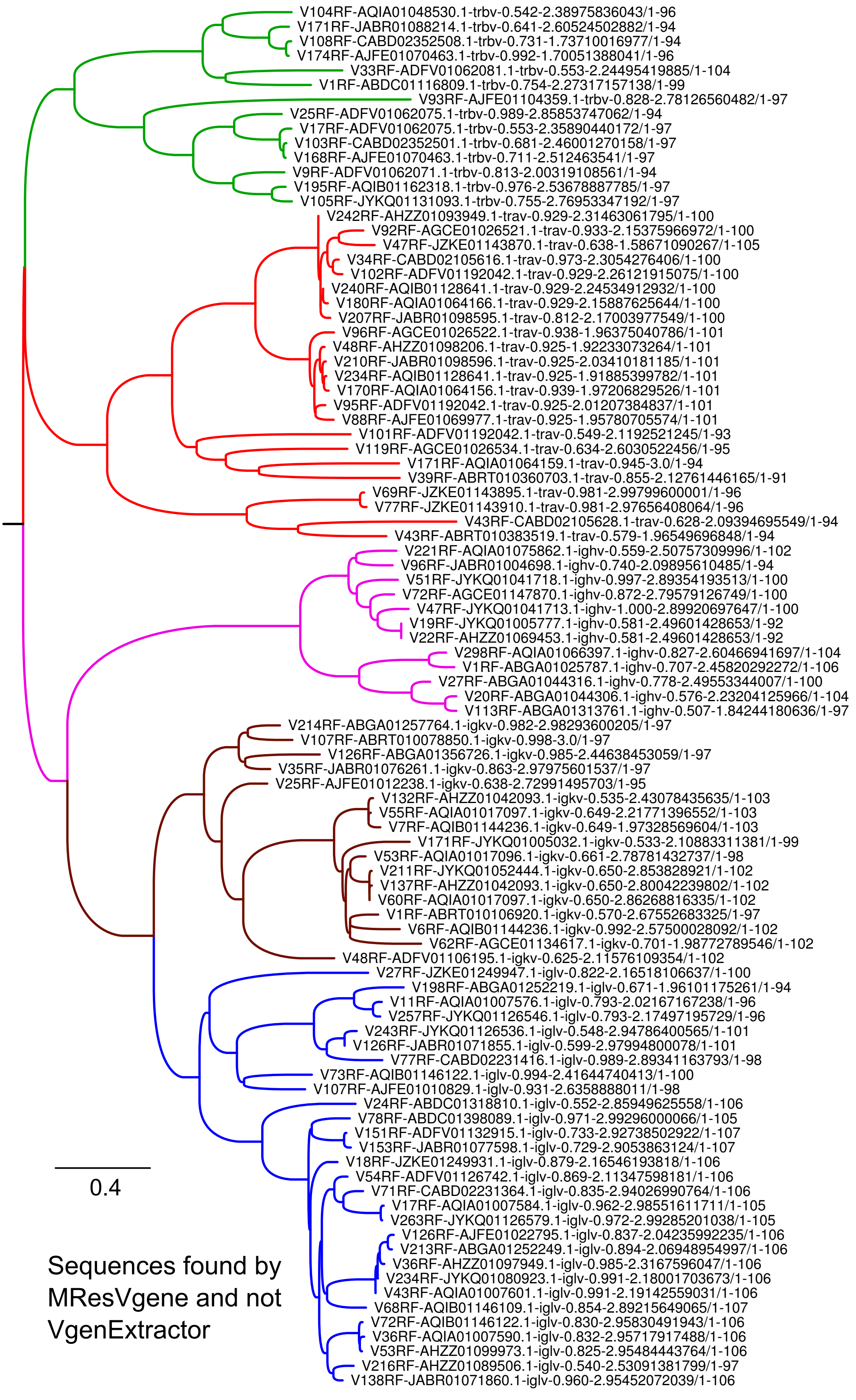

Figure S10: Phylogenetic tree of sequences discovered by MResVgene but not by VgenExtractor from 13 WGS primates.

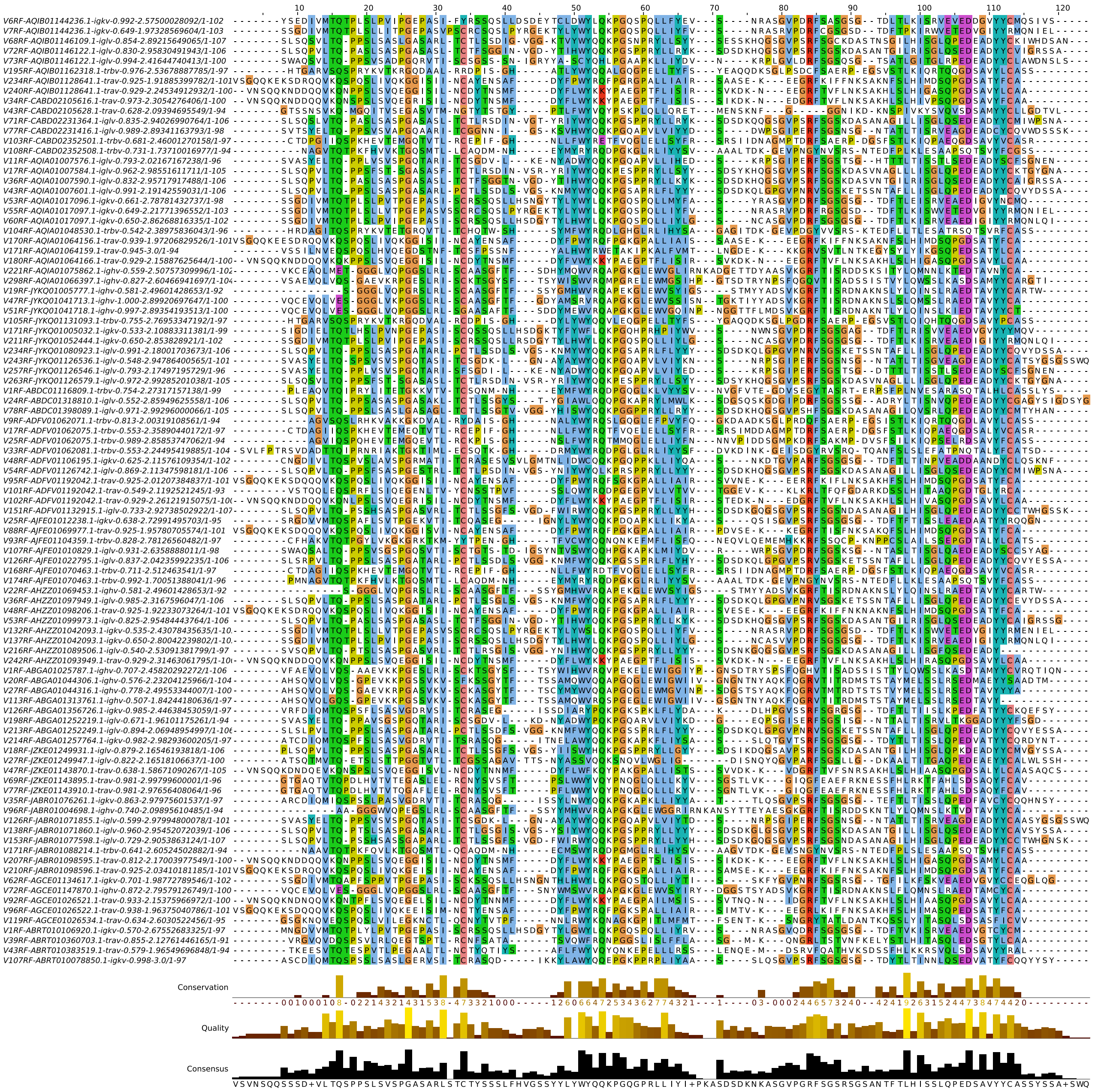

Figure S11: Alignment of sequences discovered by MResVgene but not by VgenExtractor from 13 WGS primates.

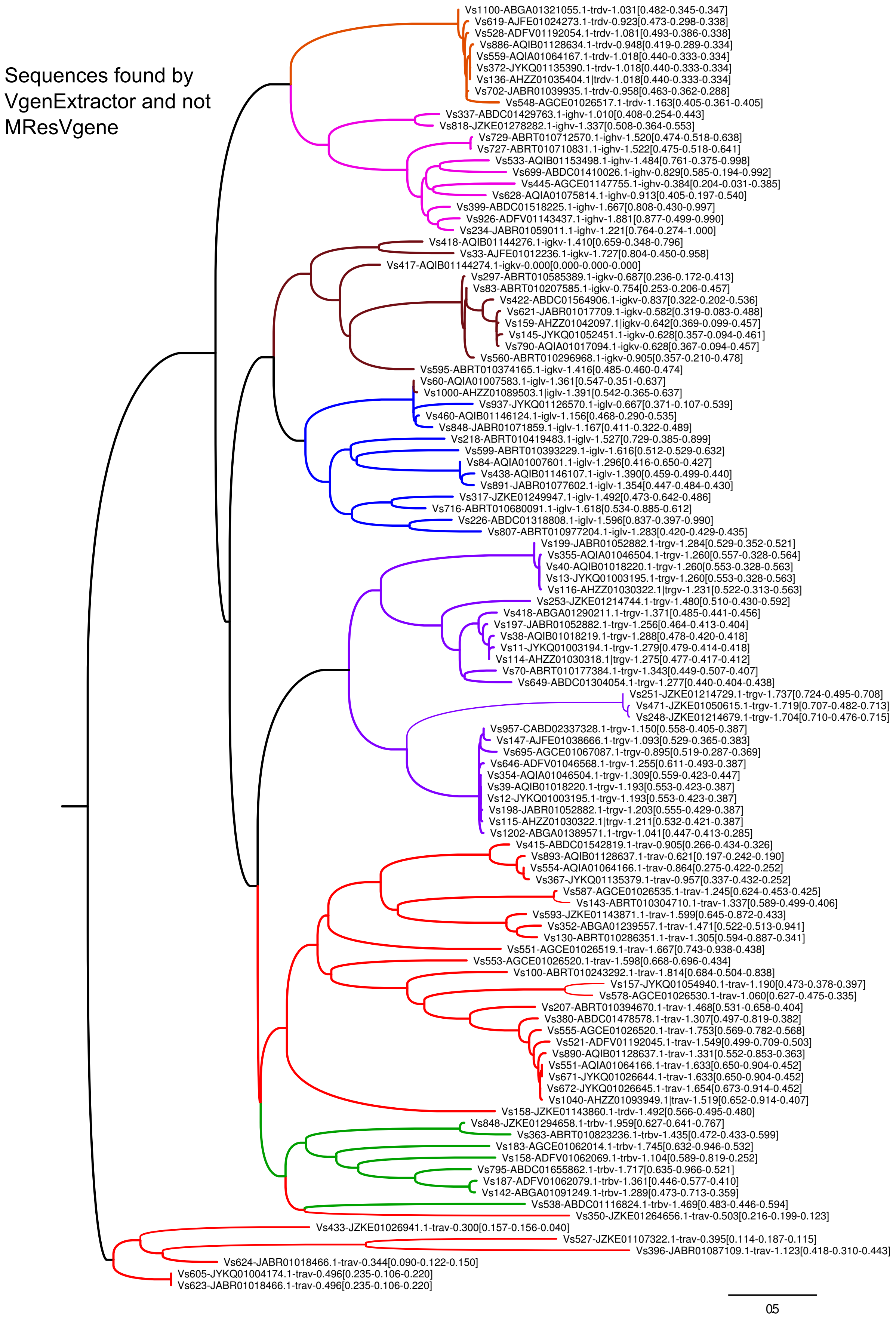

Figure S12: Phylogenetic tree of sequences discovered by VgenExtractor but not by MResVgene from 13 WGS primates.

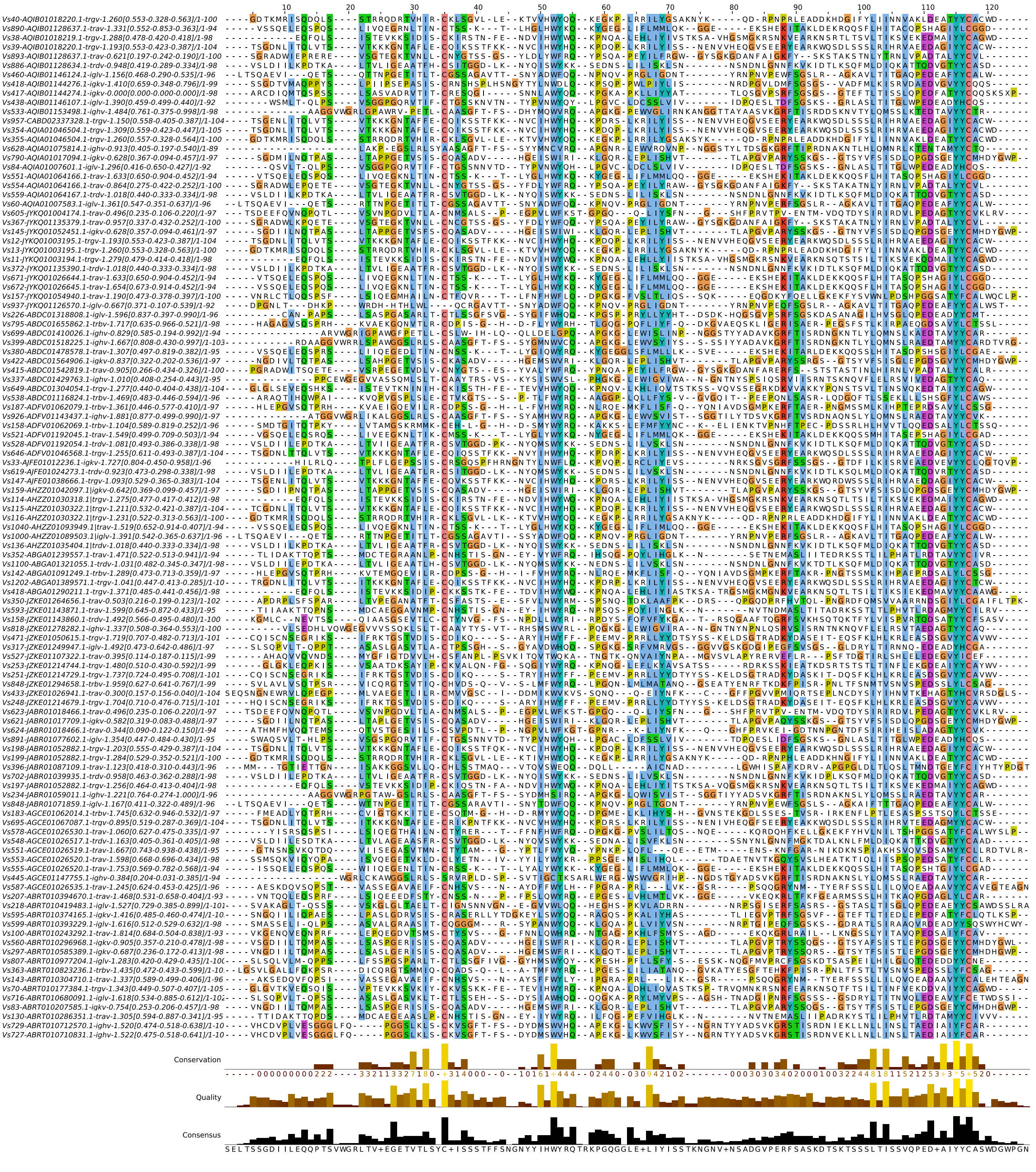

Figure S13: Alignment of sequences discovered by VgenExtractor but not by MResVgene from 13 WGS primates.

3. Comparison with ENSEMBL V-gene Annotations

Comparisons were made between MResVegene and VgenExtractor for discovering V-gene annotations from *Macaca mulatta*. Figures S14 S15 show the chromosome (and scaffold) sections for the IG and TR loci, respectively.

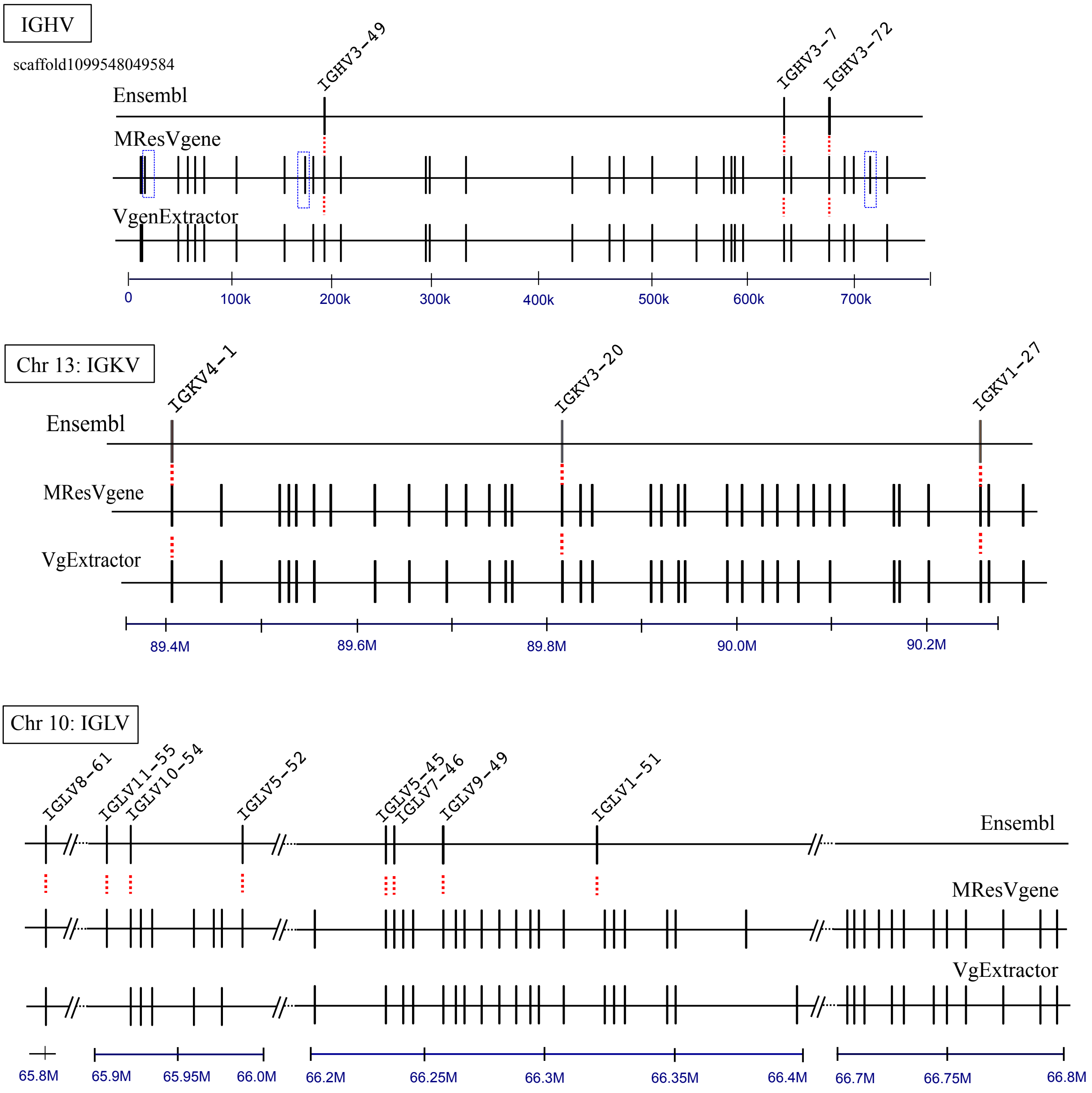

Figure S14: The IG loci Genomic annotations of the *M. mulatta* assembly (obtained from the Ensembl, ESMBL, repository) together with the MresVgene and Vgenextractor predictions.

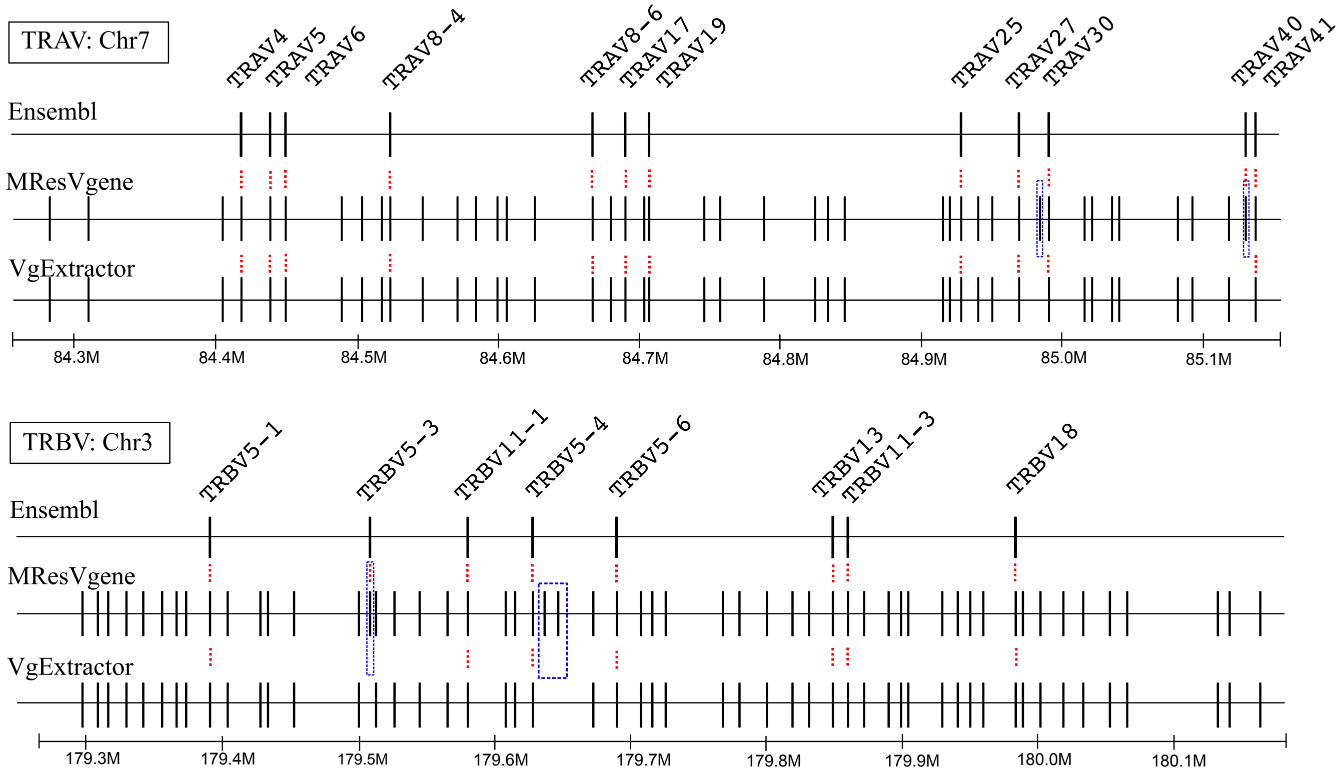

Figure S15: The TR loci Genomic annotations of the *M. mulatta* assembly (obtained from the Ensembl, ESMBL, repository) together with the MresVgene and Vgenextractor predictions.

3.1. Detailed description of Sequence difference

As seen in Figures S14 and S15, the agreement between MResVegene and VgenExtractor and ability to predict Ensembl sequence is high. The discrepencies between the two methods found in the TRAV and TRBV loci are studied in detail in Figures S16 - S19. As indicated in the manuscript, the discrepancy between MResVgene and VgenExtractor for detecting Ensembl sequences can be understood in the sequence alignments; sequences (ENS-TRAV40/1-83 and ENS-TRBV5-3/1-77) were detected by MResVgene fact but not VgenExtractor because they lack conserved motifs (i.e., ENS-TRAV40 lacks a cystein between locations 15-28 and ENS-TRBV5-3 lacks a common Y* motif in the last 15 AA).

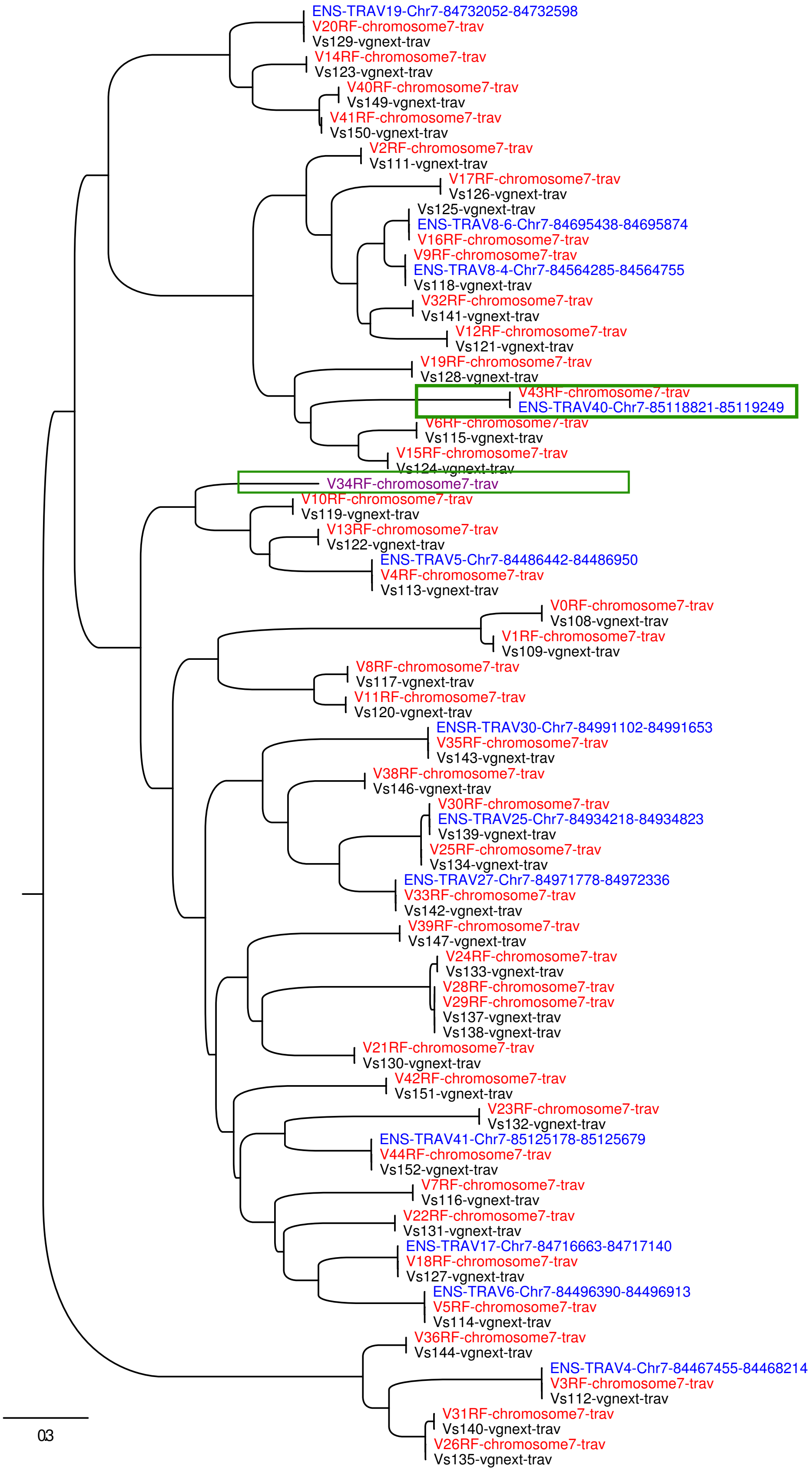

Figure S16: TRAV Tree for Ensembl comparison. Phylogenetic tree comparison of predictions from MresVgene and Vgenextractor and the the annotations of Ensembl of the TRAV locus of *M. mulatta*. V genes are obtained from the NN sequences of the Ensembl assembly.

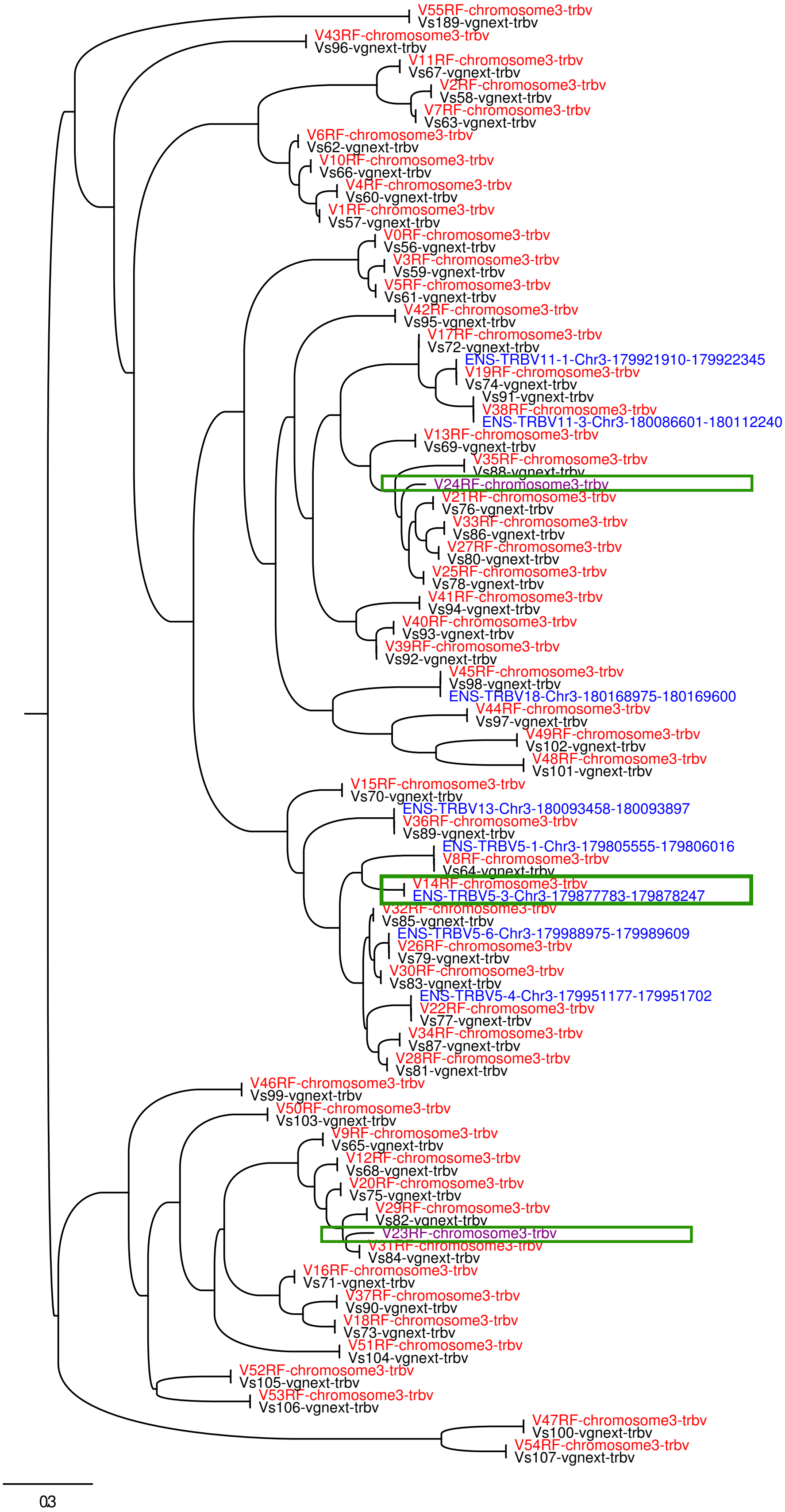

Figure S17: TRBV Tree for Ensembl comparison. Phylogenetic tree comparison of predictions from MresVgene and Vgenextractor and the the annotations of Ensembl of the TRBV locus of *M. mulatta*. V genes are obtained from the NN sequences of the Ensembl assembly.

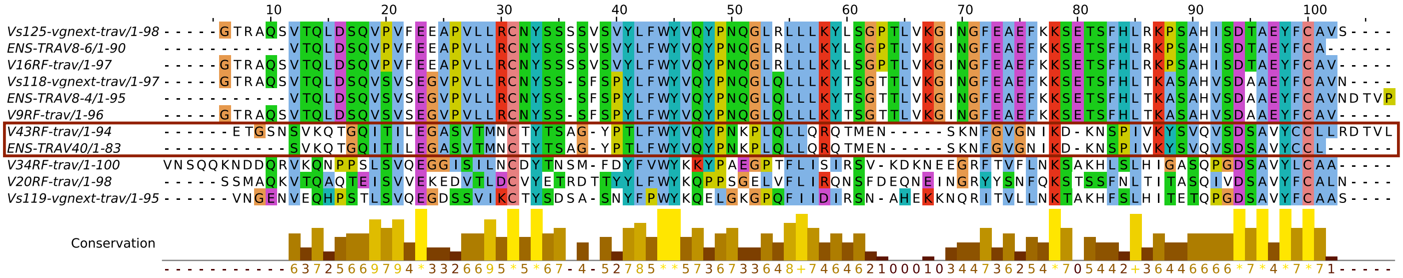

Figure S18: Alignment of TRAV sequences found by MResVgene but not by VgenExtractor for the Ensembl comparison.

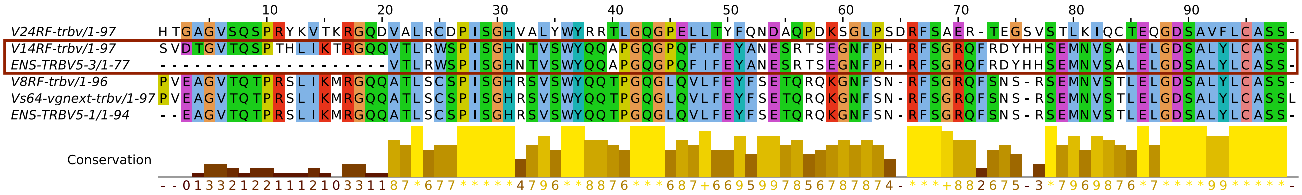

Figure S19: Alignment of TRBV sequences found by MResVgene but not by VgenExtractor for the Ensembl comparison.

The detailed chromosomes and locations for the V-genes annotated in Ensembl.


```
  "TRAV": {
     "TRAV8-6":["chr7",84695438,84695874,1],
     "TRAV40": ["chr7",85118821,85119249,1],
     "TRAV30": ["chr7",84991102,84991653,1],
     "TRAV25": ["chr7",84934218,84934823,1],
     "TRAV6":  ["chr7",84496390,84496913,1],
     "TRAV5":  ["chr7",84486442,84486950,1],
     "TRAV4":  ["chr7",84467455,84468214,1],
     "TRAV27": ["chr7",84971778,84972336,1],
     "TRAV17": ["chr7",84716663,84717140,1],
     "TRAV19": ["chr7",84732052,84732598,1],
     "TRAV8-4":["chr7",84564285,84564755,1],
     "TRAV41": ["chr7",85125178,85125679,1]
   },

   "TRBV": {
         "TRBV11-3": ["chr3", 180086601,180112240,1],
         "TRBV5-6":  ["chr3", 179988975,179989609,1],
         "TRBV5-4":  ["chr3", 179951177,179951702,1],
         "TRBV5-3":  ["chr3", 179877783,179878247,1],
         "TRBV5-1":  ["chr3", 179805555,179806016,1],
         "TRBV11-1": ["chr3", 179921910,179922345,1],
         "TRBV18":   ["chr3", 180168975,180169600,1],
         "TRBV13":   ["chr3", 180093458,180093897,1]
   },
   "IGKV": {
        "IGKV4-1": ["chr13",89436957,89437895,1],
        "IGKV1-27":["chr13",90246706,90247293,-1],
        "IGKV3-20":["chr13",89827964,89828257,-1]
   },
   "IGLV": {
      "IGLV5-52": ["chr10", 66038886,66039290, 1],
      "IGLV7-46":  ["chr10", 66222287,66222580,1],
      "IGLV9-49": ["chr10", 66252992,66253527,1],
      "IGLV1-51": ["chr10", 66335879,66336272,1],
      "IGLV5-45": ["chr10", 66226801,66227112,1],
      "IGLV11-55": ["chr10",65906638,65906946,1],
      "IGLV10-54": ["chr10",65929928,65930221,1],
      "IGLV8-61": ["chr10", 65803593,65803889,1]
   },
   "IGHV": {
       "IGHV3OR15": ["S1099214757507", 238,537,1],
       "IGHV3-23":  ["S1099214148171", 797,1845,-1],
       "IGHV3-72":  ["S1099548049584", 700393,701553,1],
       "IGHV3-7":   ["S1099548049584", 654699,654992,1],
       "IGHV3-49":  ["S1099548049584", 187218,187895,1]
   }
}
```
